# Altered Fecal Metabolomics and Potential Biomarkers of Psoriatic Arthritis Differing From Rheumatoid Arthritis

**DOI:** 10.3389/fimmu.2022.812996

**Published:** 2022-02-28

**Authors:** Nan Wang, Linjiao Yang, Lili Shang, Zhaojun Liang, Yanlin Wang, Min Feng, Shuting Yu, Xiaoying Li, Chong Gao, Zhenyu Li, Jing Luo

**Affiliations:** ^1^ Division of Rheumatology, Department of Medicine, The Second Hospital of Shanxi Medical University, Taiyuan, China; ^2^ Modern Research Center for Traditional Chinese Medicine of Shanxi University, Taiyuan, China; ^3^ Department of Pathology, Brigham and Women’s Hospital, Harvard Medical School, Boston, MA, United States

**Keywords:** feces, metabolomics, biomarker, psoriatic arthritis, UHPLC-Q-TOF-MS

## Abstract

Psoriatic arthritis (PsA) is a chronic inflammatory joint disease, and the diagnosis is quite difficult due to the unavailability of reliable clinical markers. This study aimed to investigate the fecal metabolites in PsA by comparison with rheumatoid arthritis (RA), and to identify potential diagnostic biomarkers for PsA. The metabolic profiles of the fecal samples from 27 PsA and 29 RA patients and also 36 healthy controls (HCs) were performed on ultra-high-performance liquid chromatography coupled with hybrid triple quadrupole time-of-flight mass spectrometry (UHPLC-Q-TOF-MS). And differentially altered metabolites were screened and assessed using multivariate analysis for exploring the potential biomarkers of PsA. The results showed that 154 fecal metabolites were significantly altered in PsA patients when compared with HCs, and 45 metabolites were different when compared with RA patients. A total of 14 common differential metabolites could be defined as candidate biomarkers. Furthermore, a support vector machines (SVM) model was performed to distinguish PsA from RA patients and HCs, and 5 fecal metabolites, namely, α/β-turmerone, glycerol 1-hexadecanoate, dihydrosphingosine, pantothenic acid and glutamine, were determined as biomarkers for PsA. Through the metabolic pathways analysis, we found that the abnormality of amino acid metabolism, bile acid metabolism and lipid metabolism might contribute to the occurrence and development of PsA. In summary, our research provided ideas for the early diagnosis and treatment of PsA by identifying fecal biomarkers and analyzing metabolic pathways.

## Introduction

Psoriatic arthritis (PsA) is a chronic autoimmune disease characterized by joint inflammation and skin psoriasis (1). Its effects on men and women are almost the same, with peak onset ages of 40 and 50 years, respectively (1, 2). It is a heterogeneous disease that affects multiple organ systems, namely, peripheral joints, axial joints, attachment points, skin, and nails (3, 4), which tend to be associated with obesity, metabolic syndrome, uveitis, atherosclerosis, chronic liver disease, cardiovascular disease and mental disorders (4–7). PsA is known to be affected by many aspects, namely, genetic, immune, and environmental factors, which play key roles in the development of PsA (1, 8). At present, the pathogenesis of PsA is not fully clear and its early diagnosis, and its treatment is still challenging.

Clinically, PsA is difficult to distinguish from other inflammatory joint diseases, especially rheumatoid arthritis (RA) in the early stages of onset, because their clinical presentation and manifestations have many similarities (9–12). PsA and RA are both characterized by joint pain and swelling, and have the same organ systems affected, namely, the skin, joints, eyes, vascular system, and even the immune system (12). The diagnosis of RA is mainly based on clinical symptoms and seropositivity of specific antibodies such as rheumatoid factor (RF) and anti-cyclic citrullinated peptide antibodies (anti-CCPs), while for PsA, only clinical and imaging features are helpful for the diagnosis of the disease (13). Although most patients with PsA can be differentiated from patients with RA by specific non-articular clinical features being present, and also the infrequent seropositivity for RF and anti-CCPs (13, 14). However, in clinical practice, the differential diagnosis between PsA and RA can be challenging, especially if there is a peripheral phenotype and RF or anti-CCPs are negative in about 15–20% of cases (14, 15). Therefore, new tools are needed to discover biomarkers that can be used to reliably diagnose PsA.

With the rise of new technologies for the analysis of genome, transcription, protein, metabolomics, and others, new approaches have been provided for the study of pathogenesis (16). The intestinal microbiome may affect distant sites except the intestine through immunomodulation, such as the joints (17). Recent studies have shown that metabolites in the gut play a fundamental role in the evolutionary relationship between symbiotic microorganisms and their hosts (16, 18). Metabolite profiles provide functional readings of microbial activity and can be used as intermediate phenotypes to mediate interactions between human and microorganism (19). Although studies have found that the intestinal flora has a certain influence on the occurrence and development of PsA, such as a significant increase of the Firmicutes and Actinobacteria phyla in patients with psoriatic (20), there is little evidence on the connection between the gut microbiota and psoriatic arthritis, especially their metabolites.

Metabolomics can provide information of high-throughput quantification of metabolites. It is extensively used in the research of intestinal microbiota to explore the variation of gut microbiota derived metabolites, which are closely related to the physiological and pathological processes of the host (21, 22). Nowadays, metabolomics has been widely used to discover biomarkers and key pathways related to many diseases and explain the pathological mechanisms, due to the high throughput, high sensitivity, wide coverage, and relatively low cost (21, 23, 24). Previous metabolomic studies have detected potential biomarkers of some autoimmune diseases through plasma (25), serum (26), urine (27), and synovial fluid samples (28). Previous studies have also compared the serum metabolic profiles of patients with PsA and other autoimmune diseases by a global metabolomic approach using gas chromatography time-of-flight mass spectrometry or proton nuclear magnetic resonance (14, 29). Many research groups have also focused on analyzing possible differential biomarkers between PsA and RA in synovium and serum (14, 15). However, studies on the fecal metabolism profile of PsA patients are relatively rare.

In this study, a total of 91 human fecal samples were enrolled, namely, 36 HCs, 27 PsA and 29 RA. An untargeted fecal metabolomic approach based on UHPLC-Q-TOF-MS was used to identify the differential metabolites. First, the differential metabolites between PsA and HCs, and also PsA and RA were determined. Moreover, disordered metabolic pathways in PsA patients were predicted according to differential metabolites. Finally, potential biomarkers were screened for distinguishing PsA from HCs and RA, and the biomarkers in this study showed satisfactory performance in identifying PsA.

## Materials and Methods

### Chemicals and Reagents

Analytical grade methanol and methyl tert-butyl ether (MTBE) were obtained from the Tianjin Damao chemical reagent factory. Ultrapure water was prepared using a Milli-Q water purification system (Millipore, USA). Acetonitrile (ACN), isopropanol (IPA), formic acid and ammonium acetate (LC-MS grade) were from Thermo (USA).

### Patients and Sample Collection

Between January 2019 and January 2021, 27 PsA patients, 29 RA patients (disease control) and 36 healthy controls (HCs) with matched age and gender were enrolled from the Second Hospital of Shanxi Medical University. All patients were in accord with the American College of Rheumatology (ACR) classification criteria for PsA and RA, and without the history of other autoimmune diseases. HCs also had no history of autoimmune diseases. All participants did not use probiotic diet and antibiotics in the past month, so the additional effects of gut microorganisms on intestinal metabolites were avoided. Fresh stool samples were collected from each subject within the first to two days of the hospitalization of the patient, frozen immediately, and stored at −80°C until use.

### Clinical Data Collection

Clinical data of all participants had been collected during routine laboratory assessments, namely, blood routine examination, biochemical indicators, erythrocyte sedimentation rate (ESR), C-reactive protein (CRP), rheumatoid factor (RF), anti-cyclic citrullinated peptide (anti-CCP), cytokine levels, peripheral lymphocytes and CD4^+^ T cell subset data. Blood routine examination, namely, white blood cell (WBC), hemoglobin (HGB), platelets (PLT), lymphocytes (LYM), monocytes (MON), and neutrophiles (NEU), were evaluated using the Sysmex XN-9000 automated hematology analyzer. Biochemical indicators, namely, alanine amiotransferase (ALT), aspartate aminotransferase (AST), alkaline phosphatase (ALP), glucose (GLU), urea, and creatinine (Cr), were measured using the Beckman Coulter AU680 biochemical analyzer. The levels of CRP were evaluated using the Beckman Coulter IMMAGE800 automatic protein analyzer. The quantitative detection of RF was evaluated by an enzyme-linked immunosorbent assay (ELISA), and anti-CCP was detected by an automatic chemiluminescence analyzer (KEASER 6600). Peripheral lymphocytes and CD4^+^ T cell subset were analyzed by monoclonal antibodies on a BD-FACS-CANTO II flow cytometer (Becton Dickinson, USA). The serum concentrations of IL-2, IL-4, IL-6, IL-10, IL-17, TNF-α, and IFN-γ were detected using magnetic bead-based multiplex assays (Human Th1/Th2/Th17 subpopulation test kit: Jiangxi Cellgene Biotech Co., Ltd.) following the manufacturer’s instructions.

### Sample Preparation

Polar extracts: The fecal samples were subjected to sequential solvent extraction by water and methanol according to the previous study (30). Briefly, 20 mg of lyophilized feces were weighed, and dissolved in 1 ml of ice-cold water, then vortexed and extracted by ultrasonicating in an ice bath for 20 min. The extracts were centrifuged, and the supernatant was immediately transferred. The feces were further extracted with 1 ml of ice-cold methanol for 20 min. The extracts were centrifuged, and the supernatant was immediately transferred. Then 500 μl of each supernatant was combined, and 1 ml of ice-cold methanol was added, vortexed to precipitate protein and centrifuged at 13,000 rpm for 15 min. The supernatant was evaporated to dryness using the speed vacuum concentrator. The residue was redissolved in 100 μl of methanol-water (80:20, v/v) and centrifuged at 13,000 rpm for 15 min to obtain the supernatant for metabolomic analysis.

Non-polar extracts: The fecal residue was further extracted with 1 ml of ice-cold MTBE, and then 500 μl of the supernatant was dried under a nitrogen stream. The residue was redissolved in 100 μl of isopropanol–water (70:30, v/v) and centrifuged at 13,000 rpm for 15 min, then the supernatant were transferred the for lipidomic analysis.

To ensure the stability and repeatability of the experiments, 10 μl each supernatant was pooled together as a quality control (QC) sample for both the polar and non-polar extracts, respectively.

### LC–MS Analysis

Briefly, the metabolic profiles of the fecal samples were performed on a UHPLC (ExionLCTM AD) coupled with Triple TOF 5600+ mass spectrometer (American, AB Sciex). Chromatographic separation was acquired on a Waters Acquity UPLC HSS T3 (1.8 μm, 2.1 × 100 mm). The column temperature was set at 40°C and the injected volume was 5 µl. Date acquisition was performed in full scan mode both in the positive and negative ion modes, and also coupled with information-dependent acquisition (IDA) trigger product ion scan modes. The parameters of the MS acquisition of electron spray ionization (ESI) source were listed as follows: ion spray voltage, 4,500 V in the negative ion mode and 5,500 V in the positive mode; nebulizer gas of 55 psi; heater gas of 55 psi; curtain gas of 30 psi; decluttering potential of 60 V (positive) and −60V (negative); collision energy of 35 eV (positive) and −35 eV (negative); turbo spray temperature of 550°C; the full scan range of 100–1,500 m/z and the ion scan range of 50–1,250 m/z with high sensitivity.

Polar extracts analysis: The mobile phase was consisted of 0.1% formic acid aqueous solution (A) and ACN (B) with the optimized gradient elution program as follows: 0–2 min, 2% B; 2–3.5 min, 15% B; 3.5–5 min, 15% B; 5–18 min, 60% B; 18–27 min, 60% B; 27–29 min, 95% B; 29–36 min, 95% B; 36–36.5 min, 2% B; 36.5–39 min, 2% B. The flow rate was set at 0.3 ml/min.

Non-polar extracts analysis: The mobile phase A was ACN/H_2_O (6:4) with 0.1% formic acid and the mobile phase B was IPA/ACN (9:1) mixed with 10 mM ammonium acetate. The gradient elution program was optimized as follows: 0–3 min, 32% B; 3–6 min, 45% B; 6–8 min, 52% B; 8–12 min, 58% B; 12–14 min, 66% B; 14–20 min, 70% B; 20–25 min, 75% B; 25–28 min, 99% B; 28–31 min, 99% B; 31–31.5 min, 32% B; 31.5–34 min, 32% B. The flow rate was set at 0.25 ml/min.

### Determination of Differential Metabolites by Multivariate Analysis

The raw data was imported to XCMS (version 3.6.3) for automatic data prepossessing, namely, peak picking and retention time correction. Then the resulting data matrix were imported into SIMCA 14.0 software (Umetrics, Sweden) for multivariate data analysis, namely, principal component analysis (PCA), partial least square discriminant analysis (PLS-DA), and orthogonal partial least square discriminant analysis (OPLS-DA). The variable importance in the projection (VIP) values from OPLS-DA models, fold change (FC), *t-*test (GraphPad prism 8.0) and false discovery rate (FDR, R-Studio software, version 3.6.3) correction were performed to screen the differential metabolites. The metabolites with VIP >1, FC >1.2 or FC <0.8, *p <*0.05 and FDR <0.05 were considered to be the differential metabolites, which were identified by OSI/SMMS software (Dalian ChemData Solution Information Technology Co., Ltd., PR China) and other online databases, namely, Human Metabolome Database (http://www.hmdb.ca/), Lipidmaps (https://lipidmaps.org/) and LipidBlast (https://fiehnlab.ucdavis.edu/projects/lipidblast). The differential metabolites were further presented in a heatmap with hierarchical cluster analysis (HCA) using MetaboAnalyst 5.0.

### Statistical Analysis

Statistical analysis of clinical data and differential metabolites was performed using the SPSS 22.0, Graphpad Prism 8.0 and MetaboAnalyst 5.0. Categorical and quantitative variables were described as frequencies, percentage, mean ± standard deviation or median (Q25, Q75). Data of demographic and clinical features were compared between groups by the non-parametric Mann–Whitney *U* test or Independent Sample *t-*test, as appropriate. Correlation analysis was performed using the Pearson correlation test. Receiver operating characteristic (ROC) curve analysis was used to evaluate the diagnostic performance of potential biomarkers. The support vector machines (SVM) classification model and ROC analysis for multiple biomarkers were performed by Biomarker analysis module in MetaboAnalyst 5.0.

## Results

### Demographical and Clinical Characteristics of PsA Patients

All 27 PsA patients (12 men and 15 women) were Han Chinese population (100%). Their mean age was 46.56 ± 15.04 years old. The median disease duration of PsA was 41 months (range 1–180 months). The median age at onset of PsA was 43.22 years (range 11–64 years). In 81.5% of patients with PsA, the onset began with skin manifestations, followed by joint inflammation. Among the 27 patients with PsA, 18 cases showed peripheral arthritis, 1 case showed axial arthritis, and 8 cases showed mixed peripheral spine. In terms of skin manifestations, 3 cases of PsA had no skin damage, and the remaining 24 cases had psoriatic rashes of varying degrees; In addition, 3 patients with PsA had nail lesions. All patients of PsA were newly diagnosed, with no current treatment with disease-modifying antirheumatic drugs (DMARDs) and corticosteroid for joint symptoms.

The demographics and clinical characteristics of PsA and RA patients are tabulated in **Table 1** (Additional clinical data were listed in **Supplementary Tables S1**, **S2**). There were no significant differences in age (*p* = 0.1589) and BMI (*p* = 0.6114) between the PsA and RA group by two-tailed unpaired Student’s *t*-test, and also PsA group and HC group (age *p* = 0.4750).

**Table 1:** Demographics and clinical characteristics of PsA and RA patients and health control^a^.

	PsA patients (n = 27)	RA patients (n = 29)	HC (n = 36)
Female: male	15:12	17:12	19:17
Age and years (mean ± SD)	46.56 ± 15.04	51.4 ± 9.84	49.08 ± 12.98
BMI (kg/m^2^, mean ± SD)	23.41 ± 4.20	23.93 ± 3.12	
DISEASE ACTIVITY PARAMETERS			
Age at onset (mean years, range)	43.22 (11–64)	45.62 (24–65)	
Disease duration (mean month, range)	41 (1–180)	70 (1–300)	
ESR (median mm/h, range)	21 (1–120)	46.5 (5–120)	7.5 (1–20)
CRP (median mg/, range)	7.23 (1–177)	13.35 (1–197)	
DAS28 (mean ± SD)	4.09 ± 1.31	5.25 ± 1.46	
% active (DAS28 >3.2)	20 (74.07%)	26 (89.66%)	
% remission (DAS28<2.6)	6 (22.22%)	1 (3.44%)	
SJC (mean ± SD)	3.74 ± 6.04	7.0 ± 7.44	
TJC (mean ± SD)	6.52 ± 6.73	10.17 ± 8.12	
PASI (mean ± SD)	1.54 ± 1.37	–	
AUTOANTIBODY STATUS			
RF positive, n (%)	2 (7.4%)	20 (69.0%)	
Anti- CCP positive, n (%)	1 (3.7%)	22 (75.9%)	

a ESR, erythrocyte sedimentation rate; CRP, C-reactive protein; RF, rheumatoid factor; Anti-CCP, anti-cyclic citrullinated peptide; SJC, swollen joint count; TJC, tender joint count; PASI, Psoriasis Area and Severity Index.

### Metabolic Profiling of UHPLC-Q-TOF-MS

The polar and non-polar extracts of all the fecal samples (HC, PsA, and RA groups) were analyzed by UHPLC-Q-TOF-MS. After data processing, namely, peak picking, retention time correction, and missing value filling, 12,457 and 7,622 metabolic features were detected in the positive and negative ion modes for the polar extracts, and 11,749 and 9,513 metabolic features were detected in the positive and negative ion modes for the non-polar extracts. The typical total ion chromatograms of the polar and non-polar extracts of the fecal samples are shown in **Supplementary Figure S1**.

### UHPLC-Q-TOF-MS Method Validation

Polar extracts: In order to evaluate the data quality of the metabolic profiles, a PCA model was constructed, and the repeatability of metabolic profiling was evaluated using QC samples. For the positive ion mode (**Supplementary Figure S2A**), all QC samples were tightly clustered together in the center of the PCA score plot, and fell within the 2 SD’s region and 95% confidence interval, which indicated that the analytical methods were reliable and acceptable (**Supplementary Figure S2C**). For the negative ion mode, the PCA model also showed good reliability of metabolomic platform in this study (**Supplementary Figures S2B, D**).

Non-polar extracts: In the positive and negative ion modes (**Supplementary Figure S3**), 14 QC samples were clustered closely in the PCA model and all fell within the 2 SD’s region and 95% confidence interval, which also indicated that the analytical platform provided excellent reliability required for a large-scale metabolomic study.

### Multivariate Statistical Analysis

**Figure 1:**
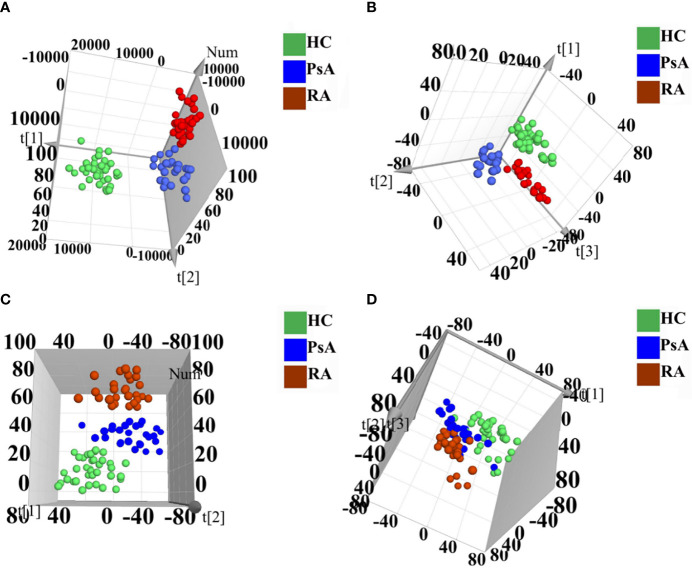
3D PLS-DA score scatter plots of polar extracts in positive ion mode **(A)** and negative ion mode **(B)**. 3D PLS-DA score scatter plots of non-polar extracts in positive ion mode **(C)** and negative ion mode **(D)**. HC group (green circle), PsA group (blue circle), and RA group (red circle).

Polar extracts: As shown in the PCA score plot of the positive ion mode (**Supplementary Figure S2A**), the PsA and RA groups showed obvious separation from HC group, whereas PsA group and RA group were overlapped. Then 3D PLS-DA was applied to further maximize their difference, in which both PsA and RA groups were separated from the HC group, and PsA and RA groups could be further separated (**Figure 1A**). In the negative ion mode, the PsA, RA, and HC groups were overlapped in the unsupervised PCA model (**Supplementary Figure S2B**), and the supervised 3D PLS-DA model could distinguish them with good separations (**Figure 1B**).

Non-polar extracts: For the PCA model, the PsA, RA, and HC groups were overlapped both in the negative and positive ion modes (**Supplementary Figures S3A, B**). While the 3D PLS-DA models could totally distinguish them in the positive ion mode, and partially distinguish them in the negative ion mode (**Figures 1C, D**).

### Differential Metabolites Between HC Group and PsA Group

**Figure 2:**
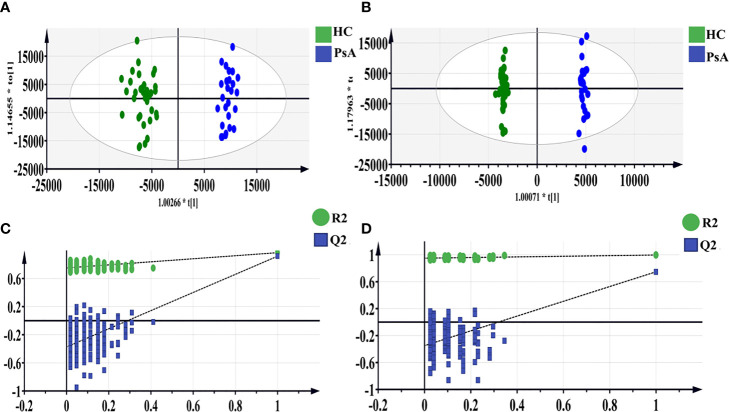
OPLS-DA score scatter plots of polar extracts between HC group and PsA group in positive **(A)** and negative ion mode **(B)**. The result of permutation test in positive ion mode **(C)** and negative ion mode **(D)**. The R^2^ and Q^2^ values were R^2^X (cum) = 0.317, R^2^Y (cum) = 0.968, Q^2^ (cum) = 0.918 in positive mode. The R^2^ and Q^2^ values were R^2^X (cum) = 0.362, R^2^Y (cum) = 0.997, Q^2^ (cum) = 0.748 in negative mode. The intercept of Q^2^ of positive and negative ion modes on the y-axis was <0, indicating a valid model.

Polar extracts: The OPLS-DA model was further constructed to determine the differential fecal metabolites between PsA and HC groups (**Figure 2**). The model parameters showed the goodness of fit and prediction ability of the OPLS-DA model both in the positive and negative ion mode (**Table 2**). Based on the criteria of VIP-values (VIP >1), *p*-value (*p <*0.05), FDR (FDR <0.05) and fold change (FC >1.2 or FC <0.8), a total of 93 differential metabolites were determined between the PsA and HC groups. Among them, 14 fecal metabolites were increased, and 79 fecal metabolites were decreased in the PsA group (**Supplementary Table S3**).

**Table 2:** The parameters of OPLS-DA model.

		*R* ^2^Y	*Q* ^2^	*p-*value
HC vs PsA	polar (ESI+)	0.968	0.918	1.50E−28
	polar (ESI−)	0.997	0.748	2.18E−11
	non-polar (ESI+)	0.962	0.525	1.19E−06
	non-polar (ESI−)	0.995	0.592	1.38E−05
RA vs PsA	polar (ESI+)	0.93	0.533	1.13E−06
	polar (ESI−)	0.969	0.169	5.25E−01
	non-polar (ESI+)	0.919	0.293	7.15E−03
	non-polar (ESI−)	0.995	0.387	2.51E−02

Non-polar extracts: The OPLS-DA model between PsA group and HC group was also constructed for the non-polar extracts with good model parameters (**Supplementary Figure S4** and **Table 2**). Based on the same criteria as described above, 61 differential metabolites were screened out. Among them, 13 fecal metabolites were increased, and 48 metabolites were decreased in the PsA patients when compared with the HCs (**Supplementary Table S3**).

### Comparison Between RA Group and PsA Group

Polar extracts: Further, the OPLS-DA model was constructed between RA group and PsA group, and model parameters also indicated the goodness of fit and prediction ability of the model for the positive ion mode (**Supplementary Figure S5** and **Table 2**). With the same criteria, 25 fecal metabolites were found higher in PsA patients, and 7 fecal metabolites were higher in RA patients (**Supplementary Table S4**). However, the model was invalid for the negative ion mode, thus no further analysis was performed (**Supplementary Figure S5**).

Non-polar extracts: Both the OPLS-DA modes in the positive and negative ion modes were valid (**Supplementary Figure S6** and **Table 2**). Thus, a total of 13 metabolites were found to express differently in PsA group compared to RA group (**Supplementary Table S4**). Among them, 8 fecal metabolites were higher in PsA patients, and 5 fecal metabolites were higher in RA patients.

### The Heatmap Analysis of RA, PsA and HC Groups

**Figure 3:**
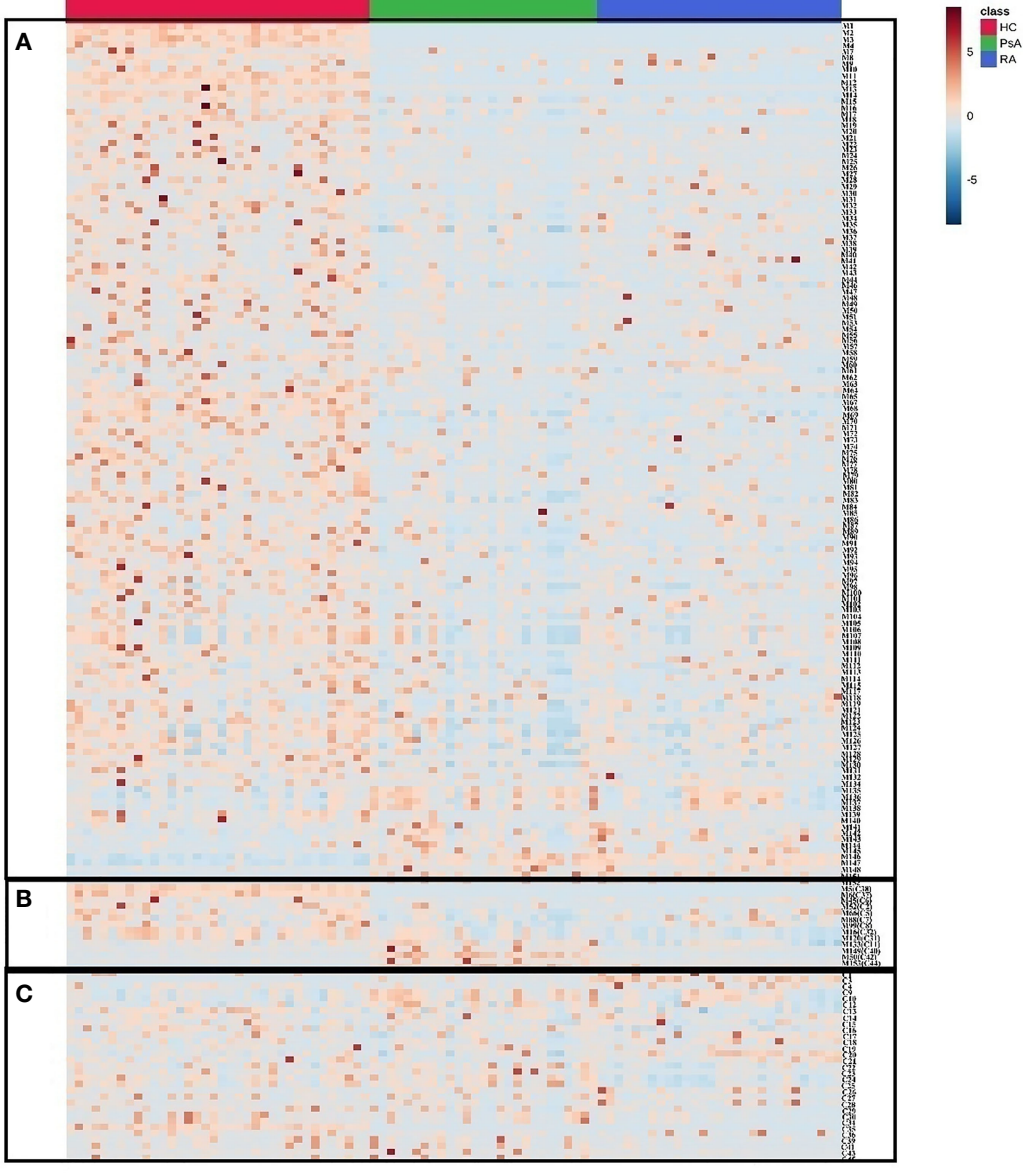
The heatmap of differential metabolites in HC, PsA and RA groups. Red color indicates high level of metabolites and blue color indicates low level of metabolites. **(A)** Metabolites distinguishing HC from PsA groups; **(B)** Metabolites distinguishing PsA from all other groups; **(C)** Metabolites distinguishing PsA from RA groups.

A heatmap was generated to provide an intuitive visualization of the content variation of the differential metabolites among 3 groups (**Figure 3** and **Supplementary Table S5**). The result was consistent with the multivariate analysis, and the HC group was obviously separated from the other groups, as most of the differential metabolites showed the highest contents in the HC group (**Figure 3**). In addition, the difference between the PsA and RA groups were also evident.

### Potential Biomarkers for PsA

**Figure 4:**
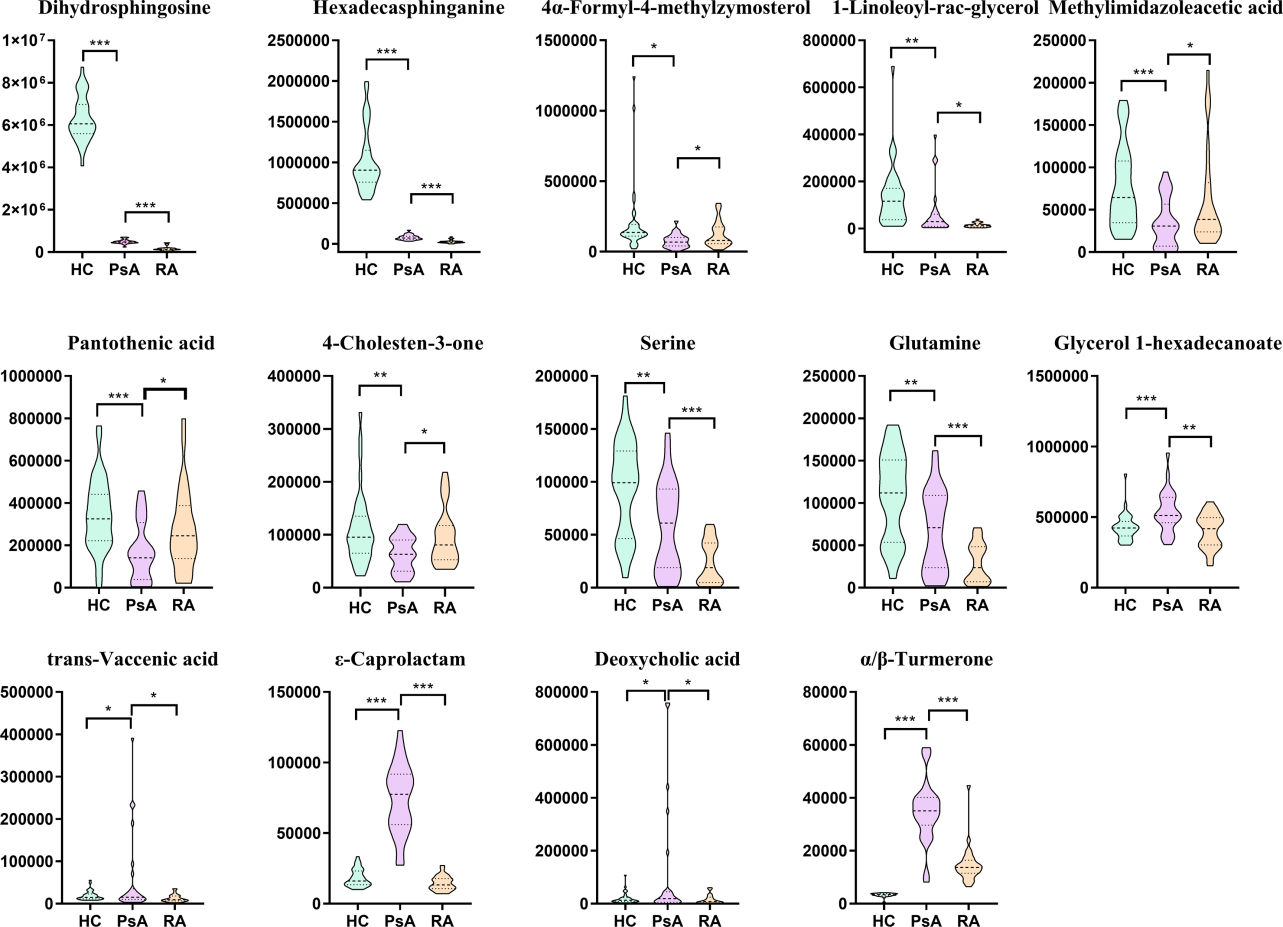
Box plots of biomarker candidates. *, **, *** denoted *p <*0.05, *p <*0.01, and *p <*0.001 in the PsA and other two groups comparison, respectively.

**Figure 5:**
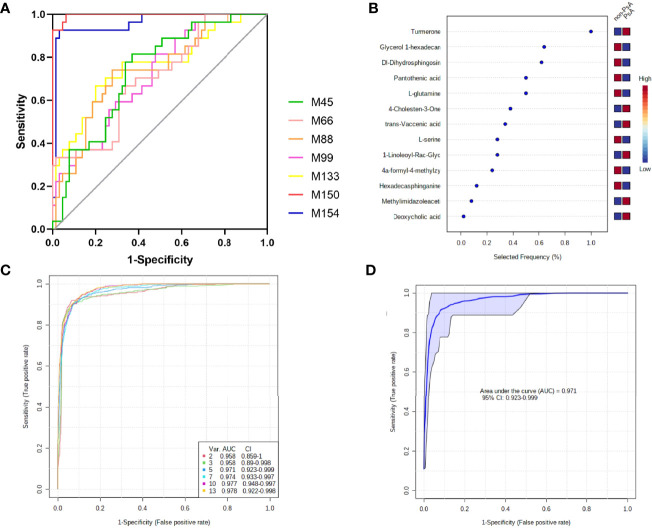
Diagnostic performance of the biomarker model for PsA diagnosis. **(A)** ROC curve analysis of 7 fecal metabolites with AUC >0.7; **(B)** The selected frequency of 13 significant features in SVM model; **(C)** SVM classifier performance for classifying PsA versus non-PsA metabolites; **(D)** ROC curve for 5 fecal microbial markers.

A total of 154 differential metabolites were screened out between PsA and HC groups, and 45 differential metabolites were found to express differently between PsA and RA groups. There were 14 common metabolites among these differential metabolites, which were defined as candidate biomarkers (**Figure 4**), namely, dihydrosphingosine, hexadecasphinganine, α/β-turmerone, ϵ-caprolactam, serine, glutamine, 4-cholesten-3-one, pantothenic acid, methylimidazoleacetic acid, vaccenic acid, deoxycholic acid, 4α-formyl-4-methylzymosterol, glycerol 1-hexadecanoate, and 1-linoleoyl-rac-glycerol. The potential discriminant biomarkers for PsA diagnosis were evaluated by ROC curve analysis to evaluate their diagnostic efficacy preliminarily (**Figure 5A** and **Supplementary Table S6**).

The areas under the ROC curve (AUCs) of 4α-formyl-4-methylzymosterol (M45), methylimidazoleacetic acid (M66), pantothenic acid (M88), 4-cholesten-3-one (M99), glycerol 1-hexadecanoate (M133), ϵ-caprolactam (M150), α/β-turmerone (M154) were 0.734, 0.704, 0.737, 0.715, 0.755, 0.996, and 0.959, respectively, for discrimination of PsA versus non-PsA (namely, HC and RA). To improve the diagnostic performance of PsA, we constructed a classifier established by Support vector machines (SVM) model in MetaboAnalyst. Since ϵ-caprolactam is an exogenous compound and cannot be synthesized in the body, it cannot be used as a biomarker and was excluded from the model. Thus, we included the 13 common differential metabolites into the SVM model. Based on the SVM classification method, the importance of the variables was ranked according to the sample weighting coefficient of the SVM analysis (**Figure 5B**). The top 2, 3, 5, 10, and 13 important features were respectively selected to build the classification/regression model, and ROC analysis on the joint model were performed (**Figure 5C**). It was found that when the top 5 metabolites in importance, namely, glycerol 1-hexadecanoate, dihydrosphingosine, pantothenic acid and glutamine, were combined for ROC analysis, the AUC reached 0.971, which had high sensitivity and specificity (**Figure 5D**). Therefore, these five metabolites have been identified as potential biomarkers that distinguish PsA patients from non-PsA.

### Differential Metabolic Pathway Analysis

**Figure 6:**
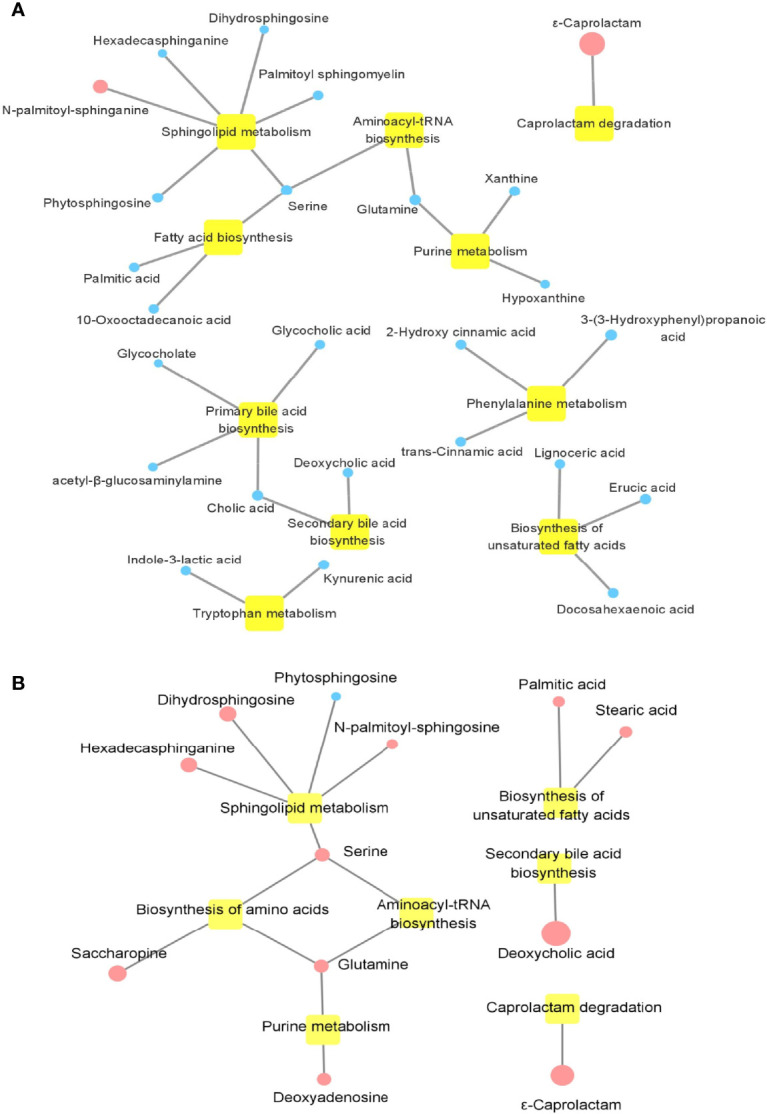
Enrichment map of metabolic pathways involved in differential metabolites. **(A)** Metabolic pathways involved in differential metabolites between PsA and HC. **(B)** Metabolic pathways involved in differential metabolites between PsA and RA. Yellow nodes represent metabolic pathways involved. Circular node represents the change in metabolite, where blue indicates a decrease in PsA, and red indicates an increase in PsA; the size of the circle indicates the fold change of metabolites in PsA compared with HC or RA.

To further explore the pathway that was possibly related with PsA, 154 different metabolites between the HCs and PsA, and 45 different metabolites between PsA and RA, respectively, were used to perform metabolic pathway analysis (**Figure 6**). As shown in **Figure 6A**, most disease-associated metabolites were decreased in PsA when compared with HCs, such as amino acid, bile acid, fatty acid, involving a variety of metabolic pathways such as amino acid metabolism, bile acid metabolism and fatty acid synthesis. When compared with RA (**Figure 6B**), the metabolic processes associated with inflammatory rheumatic diseases were altered in PsA even when clinical features were similar. The difference metabolites between PsA and RA were involved metabolic pathways, namely, sphingolipid metabolism, secondary bile acid biosynthesis, biosynthesis of unsaturated fatty acids and biosynthesis of amino acids. In addition, it was interesting that significant enrichment of ϵ-caprolactam in PsA patients, whether it is compared with HC or RA, implied the weakening of the degradation pathway of ϵ-caprolactam in PsA.

### Correlations of Metabolites With Clinical Phenotype

Compared with HCs, the ESR and neutrophils (NEU) in PsA increased (*p <*0.05). We investigated the correlations between the differentially altered metabolites and immunological parameters in patients with PsA. As indicated in **Table 3**, geranylgeraniol, lysophosphatidylethanolamine and hexadecasphinganine positively correlated with ESR and CRP. We also observed positive correlations between organic acid (namely, homovanillic acid, trans-cinnamic acid and 2-hydroxy cinnamic acid) and white blood cells (WBCs).

**Table 3:** Pearson’s correlation analysis between differentially altered metabolites and immunological parameters.

Immunological parameters	Metabolites	R	*p*- value
ESR	geranylgeraniol	0.436*	0.023
	lysophosphatidylethanolamine	0.400*	0.038
	hexadecasphinganine	0.424*	0.028
CRP	geranylgeraniol	0.531**	0.008
	lysophosphatidylethanolamine	0.423*	0.039
	hexadecasphinganine	0.557**	0.005
WBC	homovanillic acid	0.410*	0.034
	trans-cinnamic acid	0.470*	0.013
	2-hydroxy cinnamic acid	0.529**	0.005
	4-ethylphenol	0.486*	0.01
NEU	homovanillic acid	0.492**	0.009
	2-hydroxy cinnamic acid	0.561**	0.002
	4-ethylphenol	0.505**	0.007

*, ** denoted *p* < 0.05, *p* < 0.01 in differentially altered metabolites in PsA and immunological parameters comparison, respectively.

**Figure 7:**
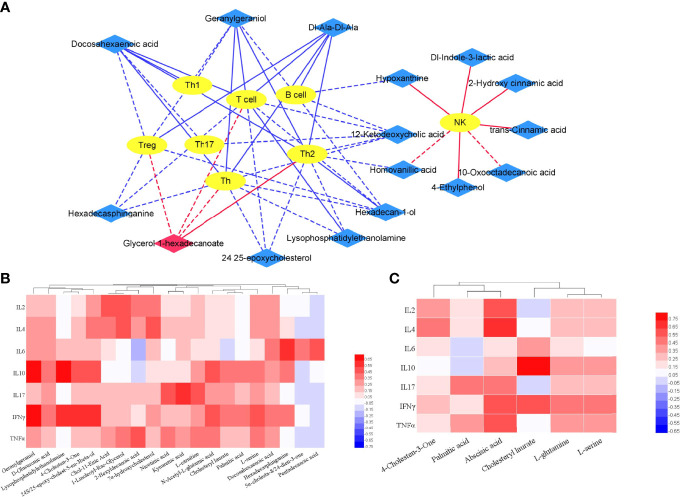
Correlation of differential metabolites and clinical features. **(A)** Integrative network of associations reflecting the interactions of differential metabolites (PsA vs HC) and lymphocyte subpopulation *via* Cytoscape (3.8.2). Network revealed both significant (*p <*0.01) and suggestive associations (0.01< *p <*0.05) between differentially abundant metabolites and lymphocyte subpopulation in PsA. Yellow oval nodes represent immune cells, red and blue diamond nodes represent the increase and decrease of metabolites in PsA, respectively. Lines connecting nodes indicate positive (red) or negative (blue) correlations. The full lines and dotted lines indicate significant correlation and suggestive correlation respectively. **(B)** Correlation of differential metabolites (PsA vs HC) and cytokines in PsA; **(C)** Correlation of differential metabolites (PsA vs RA) and Cytokines in PsA.

It was proved that T cells are heavily involved in PsA. Studies have shown that the immune system, in particular lymphocytes, has an important influence on the pathogenesis of PsA (1, 2). The correlation between the differential metabolites (PsA vs HC) and immune cell subsets was analyzed in 27 PsA patients (**Figure 7A**). Our results showed that increased glycerol 1-hexadecanoate were positively correlated with the absolute numbers of Th2 and Treg, while many decreased metabolites (such as geranylgeraniol, homovanillic acid, glycerol 1-hexadecanoate, lysophosphatidylethanolamine, malic acid and docosahexaenoic acid) were negatively correlated with the absolute numbers of B cells, Treg and T cell effector subsets Th1, Th2, and Th17. In addition, some lipids (namely, hexadecasphinganine, docosahexaenoic acid, heneicosanoic acid, and hexadecan-1-ol) exhibited negative association with Treg, and lower levels of homovanillic acid and lysophosphatidylethanolamine accompanied with increased Th2 (**Figure 7A**). The imbalance between Tregs and T cell effector subsets Th1, Th2, and Th17 might result in chronic inflammation of gut, skin or joints.

Furthermore, correlation between differential metabolites and cytokines were also calculated using Pearson correlation (**Figure 7B**). The results indicated that fecal metabolites differentially depleted in PsA showed higher correlation with cytokines of IL-6, IL-10, IL-17 and IFN-γ. For example, lysophosphatidylethanolamine in PsA was positively linked to IL-10 and IFN-γ (*p* < 0.01).

Even when symptoms of joint involvement are similar between PsA and RA, their cytokines and the metabolic processes associated with inflammatory rheumatic diseases are different. Compared with RA, the cytokines (namely, IL-2, IL-4, IL-10, IL-17, IFN-γ, and TNF-α) in PsA were significantly lower (**Supplementary Table S2**). To further understand whether characteristic metabolites of PsA contribute to disease severity, we tested for their correlations with cytokines using Pearson correlation (**Figure 7C**). We observed that the fecal metabolites differentially abundant in PsA, such as palmitic acid, abscisic acid, cholesteryl laurate, glutamine and serine, exhibited positive association with IL-2, IL-4, IL-10, IL-17, IFN-γ, and TNF-α. For example, increased abscisic acid in PsA was positively linked to IL-2 and IL-4 (*p* < 0.01).

Due to the small sample size of the PsA group, many correlations were not strong enough, the data still suggested a potential link between the fecal metabolites and clinical features of disease.

## Discussion

PsA is a chronic autoimmune disease, and its clinical features are variable and may be similar to other rheumatic diseases. It is difficult to diagnose and treat this disease early due to the lack of specificity markers. In the recent years, there has been an increasing interest to the alteration of gut microbiota in the PsA research, which has been proved to have important significance in the pathogenesis of diseases (1, 7, 20, 31). This study is the first published report on fecal metabolome of PsA to explore the potential diagnostic markers. The metabolic profiles of fecal samples might allow differentiation of PsA patients from RA patients and HCs.

A total of 154 fecal metabolites were significantly altered in PsA patients compared with the healthy controls. Interestingly, there were 45 fecal metabolites also different between PsA and RA patients. Furthermore, 14 common metabolites among these differential metabolites could be defined as candidate biomarkers. It was noteworthy that some disease-associated metabolites were altered in PsA when both compared with HCs and RA, such as amino acid, bile acid, fatty acid, vitamins and so on, involving a variety of metabolic pathways, namely, amino acid metabolism, bile acid metabolism and lipid metabolism.

### Amino Acid Metabolism

In the present study, several amino acids were altered in PsA patients compared with the HCs and RA. The levels of fecal kynurenic acid and indole-3-lactic acid were reduced in PsA patients, which are catabolites of tryptophan through the kynurenine pathway. In addition, the content of serine, glutamic acid, glutamine, and oxyproline in PsA decreased, and the content of degradation products of phenylalanine metabolism, such as 2-hydroxy cinnamic acid and 3-(3-hydroxyphenyl) propanoic acid, also decreased. The reduced glutamine was consistent with previous results found in serum of PsA (29). Differences in protein synthesis rate, immune cell consumption of glutamine and transglutaminase levels may be the reasons for the variation of glutamine levels in PsA patients (29). Other work has reported that altered amino acid concentrations may affect changes in energy metabolism (32, 33). Therefore, the data reported in this study indicated that the energy metabolism in PsA was significantly inhibited.

### Bile Acid Metabolism

The fecal primary and secondary bile acids, namely, cholic acid, nutriacholic acid, glycocholate, deoxycholic acid and 7-ketodeoxycholic acid, were depleted in PsA when compared with HCs, whereas deoxycholic acid was lower in RA. It is well known that bile acids, such as cholic acid and deoxycholic acid, are signal molecules produced by the decomposition of cholesterol through the interaction between the host and the intestinal flora, which activate bile acid activated receptors (BARs) to regulate liver lipid and glucose metabolic homeostasis and energy metabolism (34–37). Furthermore, in intestinal macrophages, G protein-coupled bile acid receptor 1 (GPBAR1) and Farnesoid-X-receptor (FXR) are highly expressed, which also occurs in innate immunity such as dendritic cells and natural killer cells (16, 35). Additionally, secondary bile acids, namely, lithocholic acid and deoxycholic acid, have been reported to protect the intestinal barrier (38, 39). Taken together, the data reported in our study indicated that alteration of bile acid homeostasis may be related to the occurrence and development of PsA.

### Lipid Metabolism

In addition to changes of amino acid and bile acid metabolism, the alteration of lipid metabolism was also highlighted in our study. Interestingly, the PsA group was characterized by altered polyunsaturated fatty acid (PUFAs) compared with non-PsA. In particular, a decrease of docosahexaenoic acid in PsA was observed. The impaired balance of PUFAs contributes to the development of some autoimmune diseases, for example, n−6/n−3 PUFAs have a major impact on the homeostasis of the immune system (40–42). Previous studies have proposed that serum PUFAs levels in patients with rheumatic diseases were usually lower (42, 43). ω-3 Fatty acids have potential immunomodulatory and anti-inflammatory effects, which could inhibit inflammation by reducing the expression of cell surface molecules and adhesion molecules, inhibiting inflammatory factors, and affecting immune cell function (44, 45). Furthermore, studies have reported that in mouse models, dietary ω-3 fatty acids had been successfully used to reduce the severity of arthritis and atopic dermatitis by promoting the differentiation of CD4^+^ T cells into Tregs (41, 46, 47).

We noticed that many metabolites (N-palmitoyl-sphinganine, phytosphingosine, dihydrosphingosine and serine) in sphingolipid metabolism pathway were altered in PsA. Sphingolipids, produced by both the host and specific bacteria, participate in specific signaling pathways of physiologic cellular functions by acting as signaling molecules or regulating the function of downstream signal molecules (16, 48). In the intestine, host sphingolipids have been thought to act directly or indirectly as inflammatory mediators (49). Sphingolipids have recently been identified as the most variable metabolite in the stool of patients with inflammatory bowel disease (IBD) (50), which was reported to be consistent with patients with PsA (51–53). Studies have shown that sphingolipid levels can regulate host immunity, and sphingolipid deficiency strains can trigger intestinal inflammation, which accompanied with an increase of IL-6 and monocyte chemoattractant protein-1 (MCP-1) in the colon (50).

In conclusion, these observations hinted that there was a potential connection between altered lipid metabolism and the pathology of PsA, which involved with sphingolipid metabolism and fatty acid metabolism at the pathway level.

### Vitamins

In addition to the metabolic changes reported above, we also found a decrease in nicotinic acid (VB3) and pantothenic acid (VB5), both of which belong to the B vitamins. These vitamins were both produced and secreted by intestinal microbes and exhibited anti-inflammatory and antioxidant activities (33, 54). Nicotinic acid is the precursor of nicotinamide adenine dinucleotide (NAD), which plays an important role in various biological processes, namely, cell metabolism, inflammatory response, aging regulation, and cell death (16). VB3 directly activates G Protein-Coupled Receptor 109A (GPCR109A), which is one of the receptor for short chain fatty acid (SCFA). In a preclinical model of colon inflammation, the activation of GPCR109A appeared to be related with the abundance of Treg in the intestinal lamina propria and the production of IL-10 (55). Moreover, VB3 reduced the secretion of inflammatory cytokines such as IL-6, TNF-α and MCP-1 when treated with lipopolysaccharide (LPS) in human monocytes (54). Pantothenic acid (VB5) is the precursor of the coenzyme A (CoA), and also an activator of fatty acid metabolism and oxidation reaction mediated by the tricarboxylic acid cycle, which is related to inflammatory homeostasis (16). In addition to the decrease in B vitamins found in PsA feces, a change in α-tocopherol (vitamin E) was also found. Vitamin E is one of the important antioxidants in the human, which can inhibit the peroxidation of lipids in cell membranes and circulate lipoproteins. It has also been reported to play an important role in the prevention and adjuvant treatment of certain chronic diseases (56).

Interestingly, we observed that ϵ-caprolactam and α/β-turmerone were uniquely increased in PsA patients. An increase of ϵ-caprolactam indicated the accumulation of xenobiotics and attenuated pathways for caprolactam degradation (ko00930) in the body. ϵ-Caprolactam is used as the raw material of nylon to produce a great deal of interior products, such as synthetic leather, synthetic fiber, plastifier and resin (57). The ubiquitous xenobiotic substances can be maintained and accumulated in the intestine due to continuous exposure to urban environment (58, 59), which might create the appropriate conditions for microbial communities in host to functionally adapt to the degradation of xenobiotic substances, including caprolactam (59–61). It was reported that oral exposure to titanium dioxide nanoparticles led to the increase of fecal caprolactam (62). In addition, deceased plasma caprolactam was also observed in the pregnant women with methylenetetrahydrofolate reductase (MTHFR) polymorphisms (63). A recent upper respiratory microbiome study found that compared with asthma patients, the abundance of caprolactam degradation pathway was significantly higher in the non-asthmatic group (61). In another recent work, the authors also found an increase in caprolactam degradation pathways in the gut microbiome of centenarians and semi-supercentenarians (59). Thus, the unique aggregation of caprolactam, which can irritate respiratory tract, eye, skin and mucous membrane (64), implied that it may have a certain effect on the occurrence of PsA, however, the exact mechanism need to be further clarified.

As regards turmerone, it is a class of polyphenolic compounds abundant in plant-based diets, obtained from turmeric (65). They are categorized as α-turmerone, ar-turmerone, and β-turmerone, and exhibit immunomodulatory effects (66). Research has shown that ar-turmerone can increase the expression level of differentiation clusters (CD40, CD80, CD83, and CD86), and subsequently induced the phenotypic and functional maturation of dendritic cells (DC). Further, the treatment with ar-turmerone can reduce the activity of acid phosphatase (ACP) in DC and promote the production of interleukin (IL-12) and TNF-α (65, 67). The human intestinal flora can metabolize polyphenols to form active substances with different functions, and polyphenols can also be transformed to affect their bioavailability (68). Therefore, the analysis of metabolic pathways is crucial for understanding these processes. However, the mechanism of how turmerone is transformed and metabolized in the intestine after entering the body to act on immune cells is still unclear. In our study, significant increase of α/β-turmerone in PsA indicated that the intestinal flora of PsA patients might lack bacteria that can metabolize polyphenols. All in all, our results indicated that the gut microbiome of PsA might be less equipped for the degradation of xenobiotics.

Actually, the fecal metabolites included the compounds from the host, microbiota and food residues, thus, not all the fecal compounds could reflect the biochemical status of the gut microbiota under the disease condition. In our study, tryptophan related metabolites, secondary bile acids, vitamins, dietary polyphenols and the degradation of xenobiotic substances were related with gut microbiota (16, 69), and thus, these metabolites implied the function of intestinal bacteria. However, the amino acids, unsaturated fatty acids and sphingomyelin were both produced by intestinal microbes and host (16), and the change of these metabolites might come from the metabolic status of the gut microbe or host.

In conclusion, we observed dysregulation of metabolic pathways in fecal samples from patients with PsA, which can be used for PsA diagnosis in a non-invasive manner. However, some limitations must be recognized in our research. First, the sample size of this study was relatively small, which may lead to missing of some differential metabolites and further limiting the generalization of the results. In the future, it is necessary to study a larger patient cohort to confirm these findings. Second, it is unclear how the intestinal microbiota affects blood characteristics due to the lack of blood samples. Further research integrating the metabolic characteristics of feces and blood is of great significance for further understanding the microbial functions in PsA. Finally, this study only conducted non-targeted metabolomics on fecal samples of PsA, and the selected candidate biomarkers lack accurate quantitative verification. Therefore, samples from multicenter cohort should be used to verify the discriminant power of biomarker panel.

In summary, this is the first metabolomic study applied to the feces of PsA and RA patients. We discovered that fecal metabolites were significantly different between these patients and HCs, and also between PsA and RA. The results might bring about the identification of new candidate biomarkers or targets for PsA diagnosis or treatment. In addition, our data emphasized that even if the clinical features were similar, the metabolic processes associated with inflammatory rheumatism differed greatly between PsA and RA. Further functional studies are necessary to explore the impact of these metabolic changes on the underlying pathogenesis mechanisms of PsA.

## Data Availability Statement

The original contributions presented in the study are publicly available. This data can be found here: http://www.ebi.ac.uk/metabolights/MTBLS4206.

## Ethics Statement

This study was approved by the ethics committee of the Second Hospital of Shanxi Medical University (2019YX266). The patients/participants provided their written informed consent to participate in this study.

## Author Contributions

JL and ZL conceived and designed the study. NW, LY, SY, and XL performed the experiments and analyzed the data. LS, ZL, YW, and MF contributed to the sample collection and storage. NW and LS were responsible for clinical data collection. NW and LY wrote the manuscript. CG, ZL, and JL reviewed and edited the manuscript. All authors listed have made a substantial, direct, and intellectual contribution to the work and approved it for publication.

## Funding

This work was supported by the Key Research and Development Project (Guide) of Shanxi Province (201803D421067), the Nature Fund Projects of Shanxi Science and Technology Department (201901D111377), the Scientific Research Project of Health commission of Shanxi Province (2019044), the Research Project Supported by Shanxi Scholarship Council of China (2020-191) and the Science and Technology Innovation Project of Shanxi Province (2020SYS08).

## Conflict of Interest

The authors declare that the research was conducted in the absence of any commercial or financial relationships that could be construed as a potential conflict of interest.

## Publisher’s Note

All claims expressed in this article are solely those of the authors and do not necessarily represent those of their affiliated organizations, or those of the publisher, the editors and the reviewers. Any product that may be evaluated in this article, or claim that may be made by its manufacturer, is not guaranteed or endorsed by the publisher.
