# Supplementary material for: Altered Fecal Metabolomics and Potential Biomarkers of Psoriatic Arthritis Differing From Rheumatoid Arthritis
Source: Front Immunol. 2022 Feb 28;13:812996. doi: 10.3389/fimmu.2022.812996 (PMC8919725; doi:10.3389/fimmu.2022.812996)
Supplement: Supplementary file 1 [file DataSheet_1.docx]

Supplementary Material

**Supplementary Table S1.** Clinical data of PsA and health controls.

|  | PsA | HC | *p*-value |
| --- | --- | --- | --- |
| **Blood routine examination** |  |  |  |
| WBC(median×10^9^/L, mean ± SD) | 6.55±2.16 | 5.29±1.13 | 0.09 |
| HGB (mediang/L, mean ± SD) | 132.81±20.04 | 143.84±14.33 | 0.019 |
| PLT(median×10^9^/L, IQR) | 258（208，314） | 229（199.5，284.5） | 0.209 |
| LYM(median×10^9^/L, IQR) | 1.98（1.39，2.32） | 1.69（1.52，2.06） | 0.828 |
| NEU (median×10^9^/L, IQR) | 3.66（2.70，4.72） | 2.82（2.34，3.64） | 0.016 |
| %LYM | 29.48±12.34 | 35.17±6.58 | 0.036 |
| % NEU | 58.83±11.92 | 55.43±6.69 | 0.189 |
| **Biochemical testing indicators** | | | |
| ALT (medianU/L, IQR ) | 16.4（11，22.6） | 17（13.4，21.45） | 0.791 |
| AST(medianU/L, IQR ) | 19.1（15.6，25.6） | 20.4（18，23.25） | 0.289 |
| GLU(medianmmol/L, IQR ) | 4.91（4.63，5.67） | 5.34（5.19，5.76） | 0.019 |
| UREA(medianmmol/L, IQR ) | 4.9（4.2，6.1） | 4.6（4.25，5.5） | 0.395 |
| Cr(medianμmol/L, IQR ) | 57（48，70） | 71（62.5，77.5） | 0.002 |
| CHO(mmol/L, mean ± SD) | 3.85±0.83 | 4.66±0.63 | 0.001 |
| TG(medianmmol/L, IQR ) | 1.14（0.79，1.46） | 1.1（0.87，1.35） | 0.886 |

**Supplementary Table S2.** Clinical data of PsA and RA.

|  | PsA | RA | *p*-value |
| --- | --- | --- | --- |
| BMI(kg/m^2^, mean ± SD) | 23.41±4.20 | 23.93±3.12 | 0.611 |
| ESR(median mm/h, IQR) | 21（13，51） | 46.5（19.5，84.0） | 0.053 |
| CRP(median mg/, IQR) | 7.23（2.0，23.62） | 13.35（3.78，70.20） | 0.163 |
| **Blood routine examination** |  |  |  |
| WBC(×10^9^/L, mean ± SD) | 6.55±2.16 | 7.47±3.17 | 0.212 |
| HGB(g/L, mean ± SD) | 132.81±20.04 | 122.59±18.67 | 0.053 |
| PLT(×10^9^/L, mean ± SD) | 283.33±111.75 | 292.55±115.00 | 0.762 |
| LYM(×10^9^/L, mean ± SD) | 1.84±0.66 | 1.73±0.75 | 0.568 |
| MON(×10^9^/L, mean ± SD) | 0.57±0.25 | 0.53±0.23 | 0.499 |
| NEU(×10^9^/L, mean ± SD) | 3.97±1.84 | 5.07±2.92 | 0.097 |
| %LYM | 29.48±12.34 | 25.16±9.98 | 0.154 |
| %MON | 9.24±4.49 | 7.48±2.57 | 0.075 |
| %NEU | 58.83±11.92 | 64.95±11.87 | 0.060 |
| **Biochemical indicators** | | | |
| ALT(medianU/L, IQR ) | 16.40（11.0, 22.60） | 16.90（10.60, 23.10） | 0.961 |
| AST(medianU/L, IQR ) | 19.10（15.60, 25.60） | 18.10（15.20, 22.10） | 0.385 |
| AST/ALT | 1.25±0.47 | 1.15±0.37 | 0.387 |
| ALP(medianU/L, IQR ) | 97（76.5, 120） | 101（82, 116） | 0.986 |
| GLU(medianmmol/L, IQR ) | 4.91（4.63, 5.67） | 5.09（4.60, 5.63） | 0.670 |
| UREA(mmol/L, mean ± SD ) | 5.10±1.22 | 5.25±191 | 0.719 |
| Cr(μmol/L, mean ± SD ) | 60.30±16.47 | 55.04±14.17 | 0.209 |
| **Lymphocyte subpopulation** | Cells/µl or % | Cells/µl or % | *p*-value |
| T cell (CD3^+^CD19^-^)( median/μL, IQR) | 1420（1029, 1705） | 1065（876, 1329） | 0.113 |
| T cell (%) | 75.61（70.70, 78.45） | 73.91（68.69, 78.06） | 0.66 |
| B cell (CD3^-^CD19^+^)( median/μL, IQR) | 197.58（143.94, 305.97） | 133.69（102.39, 213.41） | 0.077 |
| B cell (%) | 10.33（8.00，13.84） | 9.38（7.45，12.20） | 0.203 |
| Th cell (CD3^+^CD4^+^) (median/μL, IQR) | 838（640，1189） | 632（500，821） | 0.087 |
| Th (%) | 43.43（39.34，47.63） | 43.63（40.16,48.49) | 0.826 |
| CD8^+^T cell (CD3^+^CD8^+^)( median/μL, IQR) | 484 (405,627) | 415（322，570） | 0.268 |
| CD8^+^T cell (%) | 27.43（23.47，32.60） | 26.84（21.50，30.59） | 0.614 |
| NK cell (CD3^-^, CD56^+^) (median/μL, IQR) | 176.19（110.87，251.02） | 196.72 (140.23，300.32） | 0.327 |
| NK% | 10.54（7.11，16.19） | 13.81（11.71，16.64） | 0.053 |
| T+B+NK(median/μL, IQR) | 2023（1426，2448） | 1450（1191，2082） | 0.178 |
| T+B+NK (%) | 98.02（97.77，98.66） | 98.32（97.71，98.93） | 0.546 |
| **CD4+ T cell subsets** |  |  |  |
| Th1( median/μL, IQR) | 156（98.19，226.22） | 113.24（66.49，153.54） | 0.245 |
| %Th1 | 19.1（12.58，28.00） | 17.32（10.67，26.40） | 0.876 |
| Th2( median/μL, IQR) | 6.0（4.37，9.05） | 5.71（3.56，7.84） | 0.615 |
| %Th2 | 0.80（0.67，1.00） | 0.85（0.58，1.09） | 0.942 |
| Th17( median/μL, IQR) | 8.00（4.86，17.84） | 7.37（4.50，12.94） | 0.469 |
| %Th17 | 1.11（0.92，1.50） | 1.08（0.78，1.60） | 0.728 |
| Treg( median/μL, IQR) | 26.87（18.85，35.17） | 20.54（13.09，28.49） | 0.087 |
| %Treg | 3.42（2.46，4.30） | 2.83（2.29，3.78） | 0.379 |
| Th1/Th2 | 25.25（13.44，35.29） | 18.84（11.96，35.15） | 0.627 |
| Th17/Treg | 0.33（0.21，0.54） | 0.31（0.20，0.67) | 0.805 |
| Th1/Treg | 6.42 (3.28,9.51) | 6.51（3.50，10.75） | 0.621 |
| Th2/Treg | 0.25（0.16，0.34） | 0.28（0.18，0.48） | 0.394 |
| B/Treg | 7.58（6.09，8.94） | 8.61（4.45，11.90） | 0.985 |
| NK/Treg | 6.25（5.51，7.70） | 11.74（7.33，18.13） | 0.012 |
| **Cytokines** |  |  |  |
| IL-2 (median pg/mL, IQR) | 1.76（0.99，2.53） | 4.48（2.33，16.69） | 0.001 |
| IL-4 (median pg/mL, IQR) | 1.67（0.61，2.59） | 3.69（1.80，13.70） | 0.002 |
| IL-6 (median pg/mL, IQR) | 14.57（7.30，33.56） | 21.36（9.99，67.17） | 0.214 |
| IL-10 (median pg/mL, IQR) | 4.40（3.15，6.05） | 6.81（5.54，21.02） | 0.011 |
| IL-17 (median pg/mL, IQR) | 2.13（0.96，10.23） | 15.72（7.46，36.02） | 0.02 |
| IFN-γ (median pg/mL, IQR) | 4.03（2.97，5.50） | 6.62（3.66，13.76） | 0.026 |
| TNF-α (median pg/mL, IQR) | 2.58（1.55，4.49） | 5.92（2.44，47.81） | 0.04 |

**Supplementary Table S3.** Differential metabolites between HC and PsA groups.

|  | Compounds | VIP | *p* value | FDR(*p*) | FC  (PsA/HC) |
| --- | --- | --- | --- | --- | --- |
| M1 | glycocholate | 1.95 | 1.41E-26 | 4.34E-25 | 0.02 |
| M2 | palmitoylethanolamide | 3.03 | 7.80E-32 | 6.01E-30 | 0.02 |
| M3 | hexadecanamide | 1.12 | 3.72E-16 | 3.82E-15 | 0.06 |
| M4 | dihydrosphingosine | 5.96 | 1.97E-23 | 4.34E-22 | 0.07 |
| M5/C38 | dihydrosphingosine | 23.37 | 8.41E-36 | 1.29E-33 | 0.08 |
| M6/C37 | hexadecasphinganine\|C16 sphinganine | 8.84 | 1.41E-18 | 1.73E-17 | 0.08 |
| M7 | trans-9-octadecenoic acid isomer | 2.21 | 4.37E-02 | 4.48E-02 | 0.15 |
| M8 | stearamide | 1.01 | 2.52E-18 | 2.77E-17 | 0.15 |
| M9 | lysophosphatidylethanolamine(0:0/16:0) | 3.34 | 1.41E-02 | 2.19E-02 | 0.15 |
| M10 | hypoxanthine | 1.50 | 1.88E-03 | 5.17E-03 | 0.17 |
| M11 | acetyl-β-glucosaminylamine | 1.90 | 3.50E-29 | 1.35E-27 | 0.17 |
| M12 | phytenate | 1.51 | 1.46E-18 | 1.73E-17 | 0.21 |
| M13 | cis-10-nonadecenoic acid | 4.39 | 1.70E-02 | 2.49E-02 | 0.22 |
| M14 | Dl-ala-Dl-ala | 1.62 | 2.10E-30 | 1.08E-28 | 0.23 |
| M15 | hexadecan-1-ol | 1.70 | 1.39E-02 | 2.19E-02 | 0.23 |
| M16 | 3,4-epoxy-6,9-octadecadiene isomer | 1.70 | 1.39E-02 | 2.19E-02 | 0.23 |
| M17 | heneicosanoic acid | 1.88 | 1.37E-02 | 2.19E-02 | 0.24 |
| M18 | hexadecanamide | 1.18 | 5.09E-20 | 7.83E-19 | 0.24 |
| M19 | 3-methyluridine | 1.08 | 2.99E-02 | 3.60E-02 | 0.24 |
| M20 | nutriacholic acid | 5.34 | 7.12E-03 | 1.48E-02 | 0.25 |
| M21 | α-tocopherol | 2.00 | 1.18E-03 | 3.86E-03 | 0.27 |
| M22 | 5-methyluridine | 2.36 | 3.33E-02 | 3.85E-02 | 0.27 |
| M23 | cis-10-nonadecenoic acid | 2.30 | 2.43E-03 | 6.34E-03 | 0.28 |
| M24 | p-tolualdehyde | 1.36 | 1.26E-04 | 6.70E-04 | 0.28 |
| M25 | geranylgeraniol | 1.76 | 2.96E-03 | 7.05E-03 | 0.31 |
| M26 | 4α-formyl-4β-methyl-5α-cholesta-8,24-dien-3β-ol | 1.51 | 1.76E-04 | 8.74E-04 | 0.32 |
| M27 | 5β-cholestane-3α,7α,12α,26-tetraol | 1.50 | 2.66E-02 | 3.47E-02 | 0.33 |
| M28 | 7α-hydroxycholest-4-en-3-one | 1.11 | 2.84E-02 | 3.50E-02 | 0.34 |
| M29 | nutriacholic acid | 1.33 | 2.82E-02 | 3.50E-02 | 0.34 |
| M30 | glycocholic acid | 1.02 | 9.57E-04 | 3.35E-03 | 0.34 |
| M31 | (±)-abscisic acid | 1.67 | 4.84E-02 | 4.87E-02 | 0.35 |
| M32 | Indole-3-lactic acid | 1.10 | 1.44E-05 | 1.01E-04 | 0.35 |
| M33 | D-glucuronic acid | 3.96 | 1.44E-03 | 4.36E-03 | 0.35 |
| M34 | lignoceric acid | 11.99 | 3.65E-02 | 4.04E-02 | 0.36 |
| M35 | 2-phenylbutyric acid | 1.42 | 1.51E-02 | 2.28E-02 | 0.36 |
| M36 | palmitic acid | 1.06 | 7.29E-03 | 1.50E-02 | 0.36 |
| M37 | chol-11-enic acid | 4.79 | 2.44E-02 | 3.27E-02 | 0.36 |
| M38 | 10-oxooctadecanoic acid\|10-keto stearic acid | 1.45 | 1.93E-02 | 2.77E-02 | 0.36 |
| M39 | 1-linoleoyl-rac-glycerol | 6.60 | 2.20E-02 | 3.00E-02 | 0.37 |
| M40 | lysophosphatidylethanolamine(0:0/16:0) | 1.33 | 5.76E-03 | 1.23E-02 | 0.37 |
| M41 | deoxycholic acid | 1.60 | 3.79E-02 | 4.08E-02 | 0.37 |
| M42 | 5-formylsalicylic acid | 1.15 | 5.71E-03 | 1.23E-02 | 0.38 |
| M43 | 4α-carboxy-4β-methyl-5α-cholesta-8,24-dien-3β-ol | 1.70 | 2.33E-04 | 9.69E-04 | 0.38 |
| M44 | 1-palmitoyl-GPE (16:0) | 1.46 | 2.47E-03 | 6.34E-03 | 0.38 |
| M45/C6 | 4α-formyl-4β-methyl-5α-cholesta-8,24-dien-3β-ol/4α-formyl-4-methylzymosterol | 2.00 | 1.01E-02 | 1.81E-02 | 0.38 |
| M46 | 5-formylsalicylic acid | 1.11 | 2.23E-04 | 9.53E-04 | 0.39 |
| M47 | palmitoyl sphingomyelin (d18:1/16:0) | 1.18 | 1.25E-02 | 2.11E-02 | 0.39 |
| M48 | 10-undecenoic acid | 1.14 | 1.08E-02 | 1.88E-02 | 0.39 |
| M49 | 4-cholesten-3-one | 1.31 | 2.79E-02 | 3.50E-02 | 0.39 |
| M50 | 4-cholesten-3-one | 1.31 | 2.79E-02 | 3.50E-02 | 0.39 |
| M51 | dihydrosphingosine | 10.84 | 1.23E-03 | 3.86E-03 | 0.39 |
| M52/C2 | 1-linoleoyl-rac-glycerol | 1.57 | 9.98E-03 | 1.81E-02 | 0.39 |
| M53 | 10-undecenoic acid | 1.12 | 9.64E-03 | 1.77E-02 | 0.40 |
| M54 | 1-ethyladenine | 1.21 | 2.07E-02 | 2.87E-02 | 0.40 |
| M55 | 10-oxooctadecanoic acid/10-keto stearic acid isomer | 1.06 | 3.33E-02 | 3.85E-02 | 0.40 |
| M56 | aldosterone | 2.54 | 4.35E-02 | 4.48E-02 | 0.41 |
| M57 | tricosanoic acid | 5.24 | 1.32E-02 | 2.19E-02 | 0.41 |
| M58 | 9-oxoode | 1.75 | 3.84E-02 | 4.08E-02 | 0.41 |
| M59 | homovanillic acid | 1.80 | 1.65E-03 | 4.70E-03 | 0.42 |
| M60 | glycerol 1-hexadecanoate | 3.39 | 1.02E-03 | 3.51E-03 | 0.42 |
| M61 | cis-10-nonadecenoic acid | 1.41 | 3.24E-02 | 3.81E-02 | 0.43 |
| M62 | cis-10-nonadecenoic acid | 1.41 | 3.24E-02 | 3.81E-02 | 0.43 |
| M63 | trans-cinnamic acid | 1.46 | 3.72E-02 | 4.06E-02 | 0.43 |
| M64 | coenzyme Q10 | 4.20 | 1.57E-02 | 2.32E-02 | 0.43 |
| M65 | butylparaben | 1.57 | 5.57E-21 | 9.53E-20 | 0.44 |
| M66/C5 | methylimidazoleacetic acid | 1.34 | 1.61E-04 | 8.27E-04 | 0.45 |
| M67 | 24,25-epoxycholesterol | 1.22 | 1.44E-03 | 4.36E-03 | 0.46 |
| M68 | 2-hydroxy cinnamic acid | 1.11 | 3.09E-03 | 7.20E-03 | 0.46 |
| M69 | nicotinic acid | 4.58 | 1.58E-07 | 1.28E-06 | 0.46 |
| M70 | carnitine | 2.08 | 1.92E-05 | 1.28E-04 | 0.46 |
| M71 | 4α-carboxy-4β-methyl-5α-cholesta-8,24-dien-3β-ol | 1.06 | 4.46E-03 | 1.01E-02 | 0.47 |
| M72 | kynurenic acid | 1.05 | 1.85E-04 | 8.91E-04 | 0.47 |
| M73 | lysophosphatidylethanolamine(0:0/15:0) | 1.82 | 8.90E-03 | 1.71E-02 | 0.47 |
| M74 | 4-ethylphenol | 1.48 | 4.14E-02 | 4.35E-02 | 0.47 |
| M75 | delta2-THA | 1.31 | 8.59E-03 | 1.70E-02 | 0.47 |
| M76 | 4α-carboxy-4β-methyl-5α-cholesta-8,24-dien-3β-ol | 1.86 | 2.04E-04 | 8.98E-42 | 0.48 |
| M77 | 7-ketocholesterol | 1.03 | 2.02E-04 | 8.98E-04 | 0.48 |
| M78 | lysophosphatidylethanolamine(0:0/16:0) | 5.99 | 1.19E-02 | 2.08E-02 | 0.48 |
| M79 | palmitoylethanolamide | 1.82 | 3.85E-02 | 4.08E-02 | 0.48 |
| M80 | palmitoylethanolamide isomer | 1.82 | 3.85E-02 | 4.08E-02 | 0.48 |
| M81 | 4Z,7Z,10Z,13Z,16Z,19Z-docosahexaenoic acid | 2.44 | 1.94E-02 | 2.77E-22 | 0.48 |
| M82 | 7-ketodeoxycholic acid | 1.32 | 7.32E-06 | 5.37E-05 | 0.49 |
| M83 | D-(+)-malic acid | 4.11 | 3.53E-05 | 2.17E-04 | 0.49 |
| M84 | docosahexaenoic acid | 1.01 | 1.40E-02 | 1.93E-02 | 0.49 |
| M85 | stearoyl ethanolamide | 1.28 | 4.89E-02 | 4.89E-02 | 0.49 |
| M86 | deoxycholic acid | 1.40 | 2.75E-02 | 3.50E-02 | 0.50 |
| M87 | deoxycholic acid | 1.40 | 2.75E-02 | 3.50E-02 | 0.50 |
| M88/C7 | pantothenic acid | 2.70 | 7.58E-05 | 4.17E-04 | 0.51 |
| M89 | deoxycholic acid | 2.97 | 1.22E-03 | 3.86E-03 | 0.51 |
| M90 | 13-hydroxyoctadecanoic acid | 1.68 | 4.39E-02 | 4.48E-02 | 0.51 |
| M91 | Hexadecasphinganine/C16 sphinganine | 1.20 | 2.00E-04 | 8.98E-04 | 0.52 |
| M92 | D-(+)-malic acid | 3.28 | 3.86E-04 | 1.49E-03 | 0.52 |
| M93 | 4α-hydroxymethyl-4β-methyl-5α-cholesta-8,24-dien-3β-ol | 2.47 | 4.09E-03 | 9.40E-03 | 0.52 |
| M94 | mannose | 2.45 | 2.47E-02 | 3.28E-02 | 0.53 |
| M95 | xanthine | 2.11 | 2.87E-03 | 7.05E-03 | 0.53 |
| M96 | 4α-hydroxymethyl-5α-cholesta-8,24-dien-3β-ol | 1.02 | 2.96E-03 | 7.05E-03 | 0.53 |
| M97 | (±)12,13-DiHOME | 1.49 | 1.39E-02 | 2.19E-02 | 0.54 |
| M98 | stearidonic acid | 1.20 | 4.03E-04 | 1.51E-03 | 0.56 |
| M99/C8 | 4-cholesten-3-one | 1.29 | 1.60E-03 | 4.66E-03 | 0.57 |
| M100 | N-acetyl-L-glutamic acid | 1.72 | 3.23E-02 | 3.81E-02 | 0.57 |
| M101 | 1-(1Z-hexadecenyl)-sn-glycero-3-phosphoethanolamine | 2.27 | 3.57E-02 | 3.98E-02 | 0.58 |
| M102 | cholic acid | 1.59 | 4.60E-02 | 4.66E-02 | 0.58 |
| M103 | phytosphingosine | 1.38 | 2.80E-02 | 3.50E-02 | 0.58 |
| M104 | deoxycholic acid | 6.01 | 8.72E-03 | 1.70E-02 | 0.58 |
| M105 | 9-hydroxy-10E,12Z-octadecadienoic acid | 2.43 | 3.42E-02 | 3.88E-02 | 0.59 |
| M106 | erucic acid | 1.92 | 2.98E-03 | 7.05E-03 | 0.59 |
| M107 | deoxycholic acid | 14.68 | 2.70E-03 | 6.81E-03 | 0.59 |
| M108 | deoxycholic acid | 2.67 | 1.52E-03 | 4.52E-03 | 0.59 |
| M109 | γ-Tocopherol | 1.44 | 9.43E-03 | 1.75E-02 | 0.60 |
| M110 | γ-tocopherol/β-tocopherol | 1.44 | 9.43E-03 | 1.75E-02 | 0.60 |
| M111 | cholic acid | 1.42 | 1.48E-02 | 2.27E-02 | 0.60 |
| M112 | hypoxanthine | 3.25 | 3.35E-02 | 3.85E-02 | 0.60 |
| M113 | 4α-formyl-4β-methyl-5α-cholesta-8,24-dien-3β-ol | 3.04 | 1.20E-03 | 3.86E-03 | 0.61 |
| M114 | 24S,25-epoxy-cholest-5-en-3β-ol | 1.11 | 2.41E-02 | 3.26E-02 | 0.61 |
| M115 | deoxycholic acid | 1.06 | 1.56E-02 | 2.32E-02 | 0.61 |
| M116/C32 | serine | 1.11 | 1.81E-03 | 5.06E-03 | 0.62 |
| M117 | deoxycholic acid | 5.29 | 2.96E-02 | 3.59E-02 | 0.62 |
| M118 | 2-hexyldecanoic acid | 1.43 | 8.70E-03 | 1.70E-02 | 0.62 |
| M119 | glutamic acid | 3.27 | 2.04E-03 | 5.52E-03 | 0.62 |
| M120/C31 | glutamine | 1.15 | 2.44E-03 | 6.34E-03 | 0.63 |
| M121 | glutamic acid | 1.13 | 9.36E-03 | 1.75E-02 | 0.63 |
| M122 | deoxycholic acid | 8.74 | 3.39E-02 | 3.86E-02 | 0.64 |
| M123 | hypoxanthine | 2.68 | 1.49E-02 | 2.27E-02 | 0.66 |
| M124 | triamcinolone aceton | 2.48 | 1.19E-02 | 2.04E-02 | 0.67 |
| M125 | 4α-carboxy-5α-cholesta-8,24-dien-3β-ol | 1.88 | 5.07E-03 | 1.12E-02 | 0.67 |
| M126 | delta2-THA | 2.78 | 4.18E-02 | 4.35E-02 | 0.68 |
| M127 | oxoproline/pyroglutamic acid | 1.90 | 2.60E-02 | 3.43E-02 | 0.68 |
| M128 | deoxycholic acid | 3.36 | 8.42E-03 | 1.70E-02 | 0.69 |
| M129 | 3-(3-hydroxyphenyl)propanoic acid | 2.49 | 1.33E-02 | 2.19E-02 | 0.72 |
| M130 | 12-ketodeoxycholic acid | 2.05 | 7.77E-04 | 2.78E-03 | 0.76 |
| M131 | Bis(2-ethylhexyl)-phthalate | 2.05 | 7.77E-04 | 2.78E-03 | 0.76 |
| M132 | glycidyl stearate | 1.57 | 2.96E-02 | 3.59E-02 | 1.24 |
| M133/C11 | glycerol 1-hexadecanoate | 2.22 | 3.02E-04 | 1.19E-03 | 1.28 |
| M134 | glycerol 1-hexadecanoate | 2.22 | 3.02E-04 | 1.19E-03 | 1.28 |
| M135 | MG(0:0/18:3(9Z,12Z,15Z)/0:0) isomer | 4.14 | 1.86E-06 | 1.43E-05 | 1.31 |
| M136 | Hexadecasphinganine/C16 sphinganine | 14.20 | 2.81E-05 | 1.80E-04 | 1.39 |
| M137 | N-palmitoyl-sphinganine (d18:0/16:0) | 7.37 | 2.02E-02 | 2.85E-02 | 1.47 |
| M138 | dihydrosphingosine | 8.86 | 4.96E-05 | 2.83E-04 | 1.52 |
| M139 | dihydrosphingosine | 8.86 | 4.96E-05 | 2.83E-04 | 1.52 |
| M140 | deoxycholic acid | 4.08 | 4.16E-02 | 4.35E-02 | 1.55 |
| M141 | 5α-cholesta-8,24-dien-3-one | 1.41 | 3.69E-02 | 4.06E-02 | 1.56 |
| M142 | palmitic acid | 3.62 | 4.75E-03 | 1.06E-02 | 1.65 |
| M143 | pentadecanoic acid | 1.47 | 1.05E-02 | 1.87E-02 | 1.66 |
| M144 | dihydrosphingosine | 3.17 | 2.18E-02 | 3.00E-02 | 1.85 |
| M145 | 4-cholesten-3-one | 1.05 | 6.94E-03 | 1.46E-02 | 2.02 |
| M146 | palmitic acid | 1.01 | 3.21E-13 | 2.91E-12 | 2.35 |
| M147 | glycerol 1-hexadecanoate | 3.29 | 2.40E-23 | 4.63E-22 | 2.65 |
| M148 | 3,12 diketocholanic acid | 3.30 | 3.49E-02 | 3.92E-02 | 2.83 |
| M149/C40 | trans-vaccenic acid | 1.07 | 2.05E-02 | 2.87E-02 | 2.96 |
| M150/C42 | ε-caprolactam | 2.14 | 6.45E-20 | 9.04E-19 | 4.07 |
| M151 | cholesteryl laurate | 2.05 | 3.23E-11 | 2.76E-10 | 4.36 |
| M152 | palmitic acid | 2.37 | 1.37E-14 | 1.32E-13 | 4.79 |
| M153/C44 | deoxycholic acid | 1.63 | 1.79E-02 | 2.60E-02 | 5.21 |
| M154/C33 | α/β-turmerone | 1.67 | 1.96E-24 | 5.03E-23 | 10.49 |

**Supplementary Table S4.** Differential metabolites between RA and PsA groups.

|  | Compounds | VIP | *p* value | FDR(*p*) | FC  (PsA/RA) |
| --- | --- | --- | --- | --- | --- |
| C1 | abietic acid | 1.57 | 3.80E-02 | 4.50E-02 | 0.22 |
| C2/M52 | 1-linoleoyl-rac-glycerol | 1.68 | 1.72E-02 | 2.69E-02 | 0.24 |
| C3 | cholesterol | 1.80 | 2.79E-06 | 1.57E-05 | 0.37 |
| C4 | cholesterol | 1.52 | 4.40E-02 | 4.95E-02 | 0.45 |
| C5/M66 | methylimidazoleacetic acid | 1.70 | 3.27E-02 | 4.33E-02 | 0.56 |
| C6/M45 | 4α-formyl-4β-methyl-5α-cholesta-8,24-dien-3β-ol/4α-formyl-4-methylzymosterol | 1.58 | 1.66E-02 | 2.69E-02 | 0.60 |
| C7/M88 | pantothenic acid | 1.71 | 4.18E-02 | 4.82E-02 | 0.65 |
| C8/M99 | 4-cholesten-3-one | 1.78 | 1.02E-02 | 2.09E-02 | 0.67 |
| C9 | dihydrosphingosine | 10.84 | 1.23E-03 | 3.45E-03 | 0.70 |
| C10 | phytosphingosine | 12.35 | 4.57E-03 | 1.14E-02 | 0.75 |
| C11/M133 | glycerol 1-hexadecanoate | 3.39 | 1.02E-03 | 3.07E-03 | 0.75 |
| C12 | MG(0:0/18:3(9Z,12Z,15Z)/0:0) isomer | 1.04 | 3.72E-02 | 4.50E-02 | 0.79 |
| C13 | methyl cinnamate | 2.87 | 4.91E-05 | 2.20E-04 | 1.23 |
| C14 | ricinoleic acid | 2.08 | 4.21E-02 | 4.21E-02 | 1.23 |
| C15 | N-palmitoyl-sphingosine (d18:1/16:0) | 1.05 | 4.68E-02 | 4.99E-02 | 1.31 |
| C16 | erucamide | 3.84 | 2.83E-08 | 2.12E-07 | 1.48 |
| C17 | palmitic acid | 1.85 | 3.43E-03 | 9.08E-03 | 1.51 |
| C18 | stearic acid | 1.39 | 3.77E-02 | 4.50E-02 | 1.74 |
| C19 | abscisic acid | 2.08 | 1.65E-02 | 2.69E-02 | 1.75 |
| C20 | cholesteryl laurate | 1.21 | 5.38E-05 | 2.20E-04 | 1.78 |
| C21 | coenzyme Q10 | 2.50 | 2.03E-02 | 3.05E-02 | 1.80 |
| C22 | stearamide | 2.56 | 9.57E-05 | 3.59E-04 | 1.82 |
| C23 | deoxycholic acid | 2.00 | 7.35E-03 | 7.51E-03 | 1.88 |
| C24 | oleamide | 3.26 | 7.99E-07 | 5.14E-06 | 1.94 |
| C25 | hexadecanamide | 2.83 | 9.46E-06 | 4.73E-05 | 2.07 |
| C26 | 4,8,12,15,19,21-tetracosahexaenoic acid | 5.02 | 4.70E-02 | 4.99E-02 | 2.08 |
| C27 | 3,4-epoxy-6,9-octadecadiene | 2.30 | 1.73E-02 | 2.69E-02 | 2.09 |
| C28 | deoxycholic acid | 1.60 | 3.79E-02 | 4.50E-02 | 2.14 |
| C29 | deoxyadenosine | 2.43 | 6.78E-03 | 1.53E-02 | 2.16 |
| C30 | farnesylacetone | 1.90 | 2.65E-02 | 3.61E-02 | 2.30 |
| C31/M120 | glutamine | 3.41 | 2.60E-04 | 9.00E-04 | 2.31 |
| C32/M116 | serine | 3.80 | 2.82E-04 | 9.05E-04 | 2.38 |
| C33/M154 | α/β-Turmerone | 5.84 | 1.57E-10 | 1.76E-09 | 2.40 |
| C34 | farnesylacetone | 1.81 | 2.58E-02 | 3.61E-02 | 2.52 |
| C35 | dihydrosphingosine | 7.50 | 2.92E-11 | 4.38E-10 | 2.66 |
| C36 | MG(0:0/18:3(9Z,12Z,15Z)/0:0) | 1.21 | 4.76E-02 | 4.99E-02 | 2.73 |
| C37/M6 | Hexadecasphinganine/C16 sphinganine | 6.66 | 9.47E-10 | 8.53E-09 | 2.91 |
| C38/M5 | dihydrosphingosine | 9.00 | 5.20E-16 | 1.17E-14 | 3.06 |
| C39 | saccharopine | 2.57 | 1.37E-02 | 2.54E-02 | 3.37 |
| C40/M149 | trans-vaccenic acid | 2.14 | 1.41E-02 | 2.54E-02 | 4.57 |
| C41 | saccharopine | 3.30 | 5.22E-03 | 1.24E-02 | 4.83 |
| C42/M150 | ε-caprolactam | 5.09 | 1.83E-17 | 8.22E-16 | 5.05 |
| C43 | 3,4-epoxy-6,9-octadecadiene | 2.15 | 1.29E-02 | 2.53E-02 | 5.12 |
| C44/M153 | deoxycholic acid | 2.09 | 2.43E-02 | 3.53E-02 | 6.79 |
| C45 | maltitol | 2.25 | 1.00E-02 | 2.09E-02 | 7.12 |

**Supplementary Table S6.** The diagnostic performance of the 14 common metabolites.

|  | Metabolite | AUC | 95% CI | Sensitivity | Specificity | Cutoffs |
| --- | --- | --- | --- | --- | --- | --- |
| M5 | dihydrosphingosine | 0.435 | 0.317-0.554 | 1 | 0.385 | 0.385 |
| M6 | hexadecasphinganine | 0.418 | 0.301-0.535 | 1 | 0.369 | 0.369 |
| M45 | 4α-formyl-4-methylzymosterol | 0.734 | 0.629-0.840 | 0.815 | 0.631 | 0.446 |
| M52 | 1-linoleoyl-rac-glycerol | 0.436 | 0.306-0.565 | 0.111 | 0.938 | 0.049 |
| M66 | methylimidazoleacetic acid | 0.704 | 0.589-0.819 | 0.667 | 0.662 | 0.329 |
| M88 | pantothenic acid | 0.737 | 0.625-0.849 | 0.741 | 0.723 | 0.464 |
| M99 | 4-cholesten-3-one | 0.715 | 0.606-0.824 | 0.815 | 0.523 | 0.338 |
| M116 | serine | 0.474 | 0.349-0.600 | 0.519 | 0.6 | 0.119 |
| M120 | glutamine | 0.482 | 0.356-0.608 | 0.519 | 0.585 | 0.104 |
| M133 | glycerol 1-hexadecanoate | 0.755 | 0.640-0.870 | 0.667 | 0.8 | 0.467 |
| M149 | trans-vaccenic acid | 0.585 | 0.443-0.727 | 0.222 | 1 | 0.222 |
| M150 | ε-caprolactam | 0.996 | 0.989-1.0 | 1 | 0.938 | 0.938 |
| M153 | deoxycholic acid | 0.582 | 0.439-0.725 | 0.444 | 0.8 | 0.244 |
| M154 | α/β-Turmerone | 0.959 | 0.913-1.0 | 0.926 | 0.969 | 0.895 |


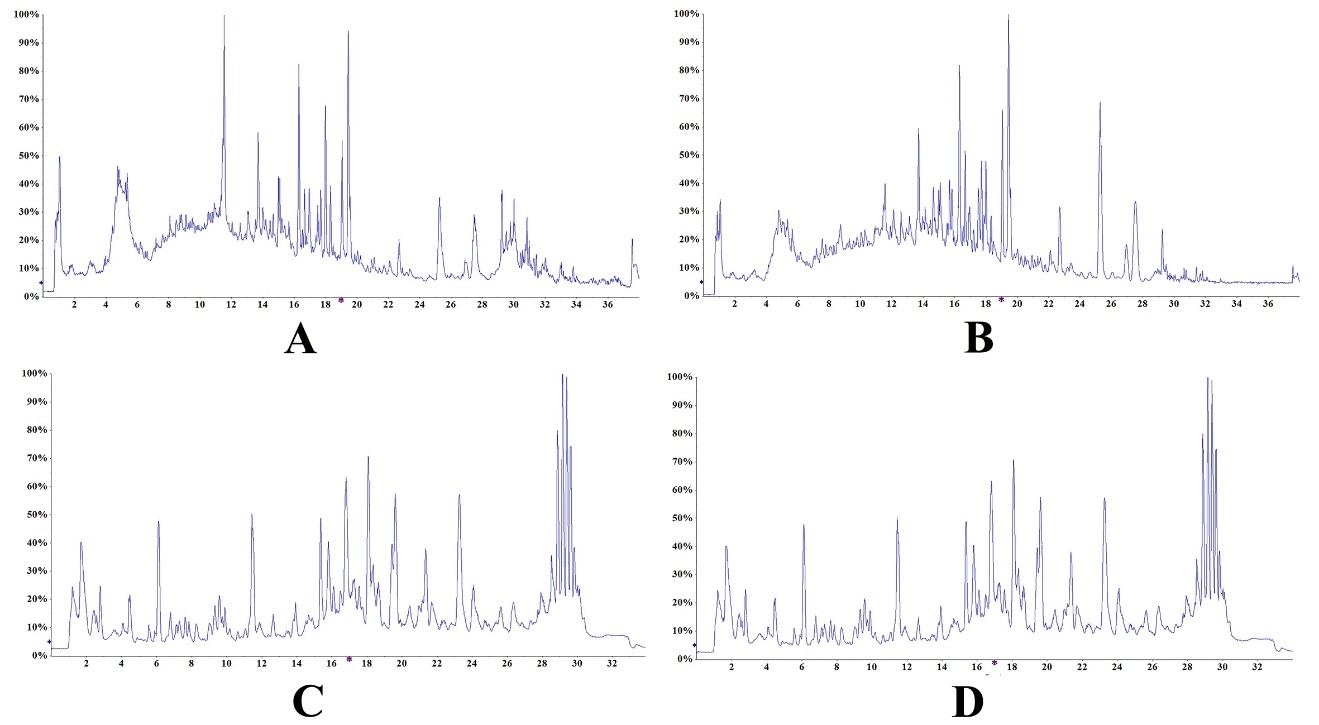


**Supplementary Figure S1.** The typical total ion chromatograms of the polar extracts in positive (A) and negative (B) ion modes. The typical total ion chromatograms of the non-polar extracts in positive (C) and negative (D) ion modes.


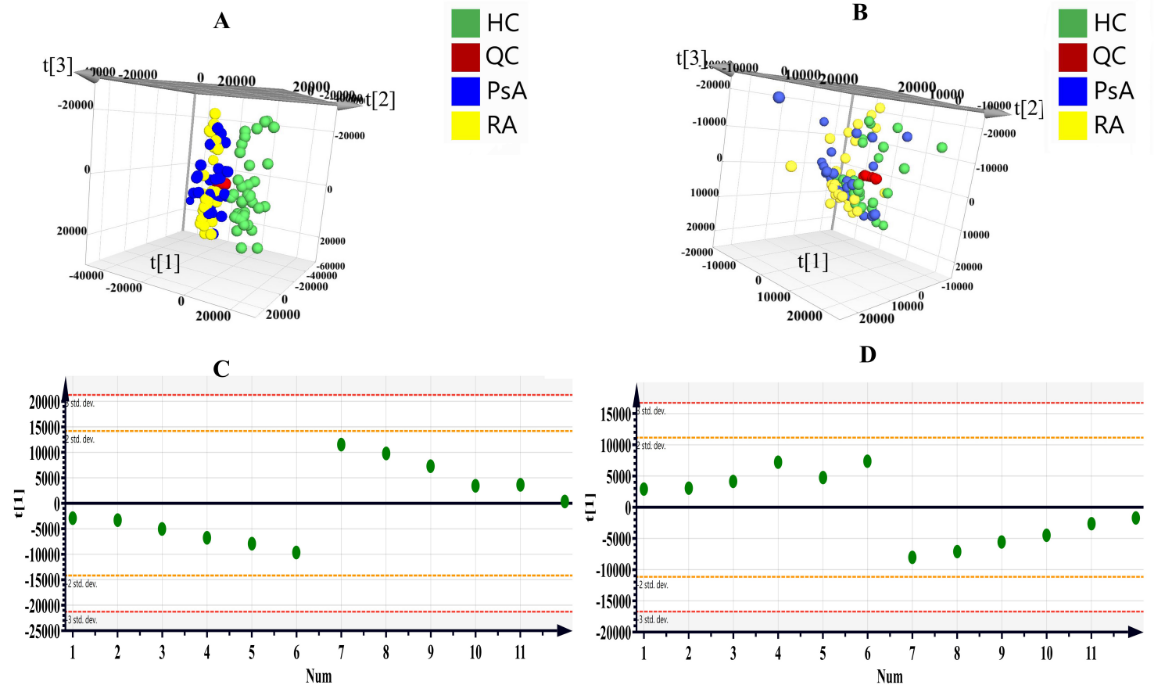


**Supplementary Figure S2.** The PCA score plot of polar extracts in the positive (A) and negative ion mode (B). The PCA line score plots of quality control (QC) samples in positive (C) and negative ion mode (D). HC group (green circle), PsA group (blue circle), and RA group (yellow circle), QC samples (red circle).


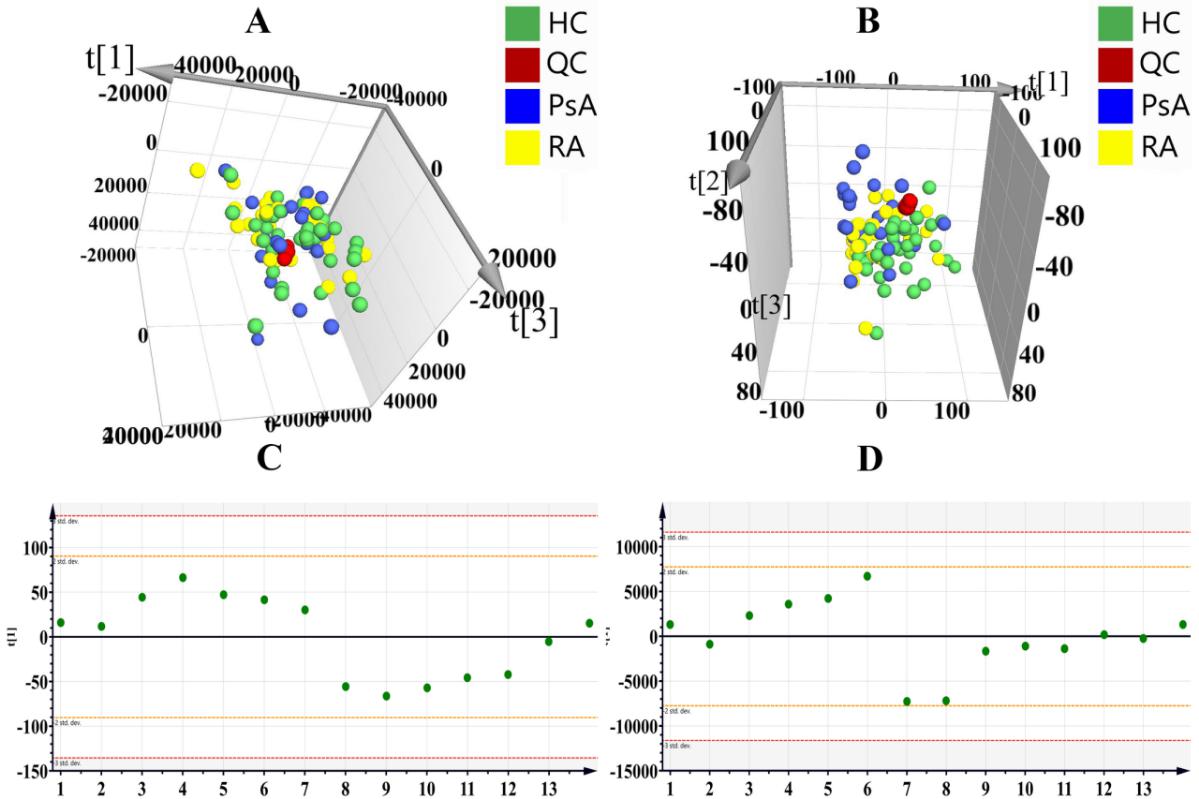


**Supplementary Figure S3.** The PCA score plot of non-polar extracts in the positive (A) and negative ion mode (B). The PCA line score plots of quality control (QC) samples in positive (C) and negative ion mode (D). HC group (green circle), PsA group (blue circle), and RA group (yellow circle), QC samples (red circle).


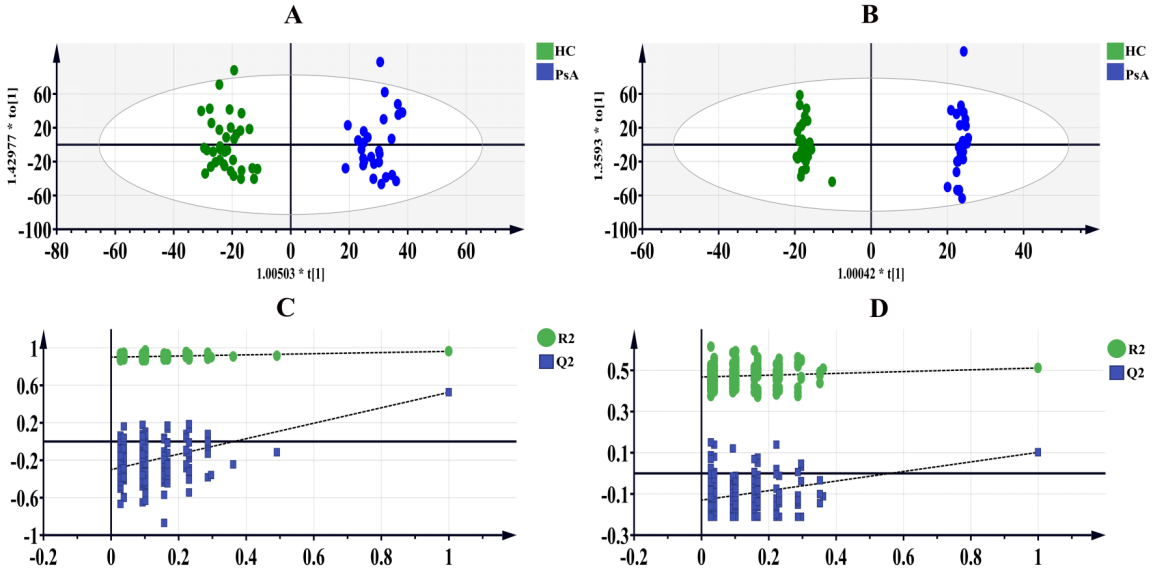


**Supplementary Figure S4.** OPLS-DA score scatter plots of non-polar extracts between HC group and PsA group in positive ion mode (A) and negative ion mode (B). The result of permutation test in positive ion mode (C) and negative ion mode (D). The R^2^and Q^2^values were R^2^X (cum) = 0.284, R^2^Y (cum) = 0.962, Q^2^ (cum) = 0.525 in positive mode. The R^2^ and Q^2^ values were R^2^X (cum) = 0.398, R^2^Y (cum) = 0.995, Q^2^ (cum) = 0.592 in negative mode.


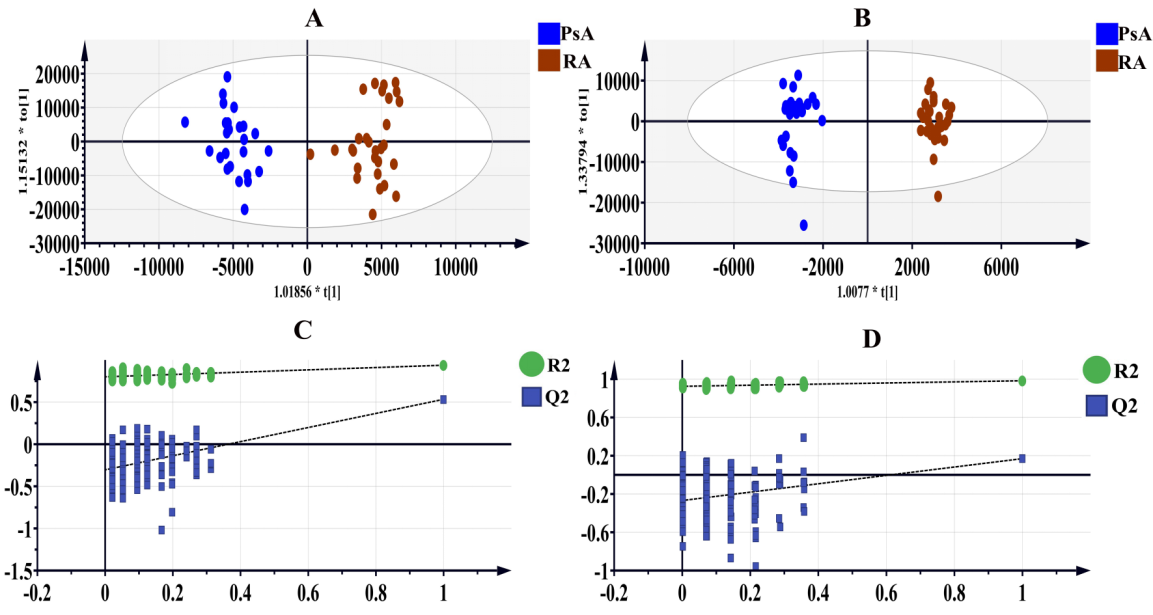


**Supplementary Figure S5.** OPLS-DA score scatter plots of polar extracts between PsA group and RA group in positive ion mode (A) and negative ion mode (B). The result of permutation test in positive ion mode (C) and negative ion mode (D). The R^2^ and Q^2^ values were R^2^X (cum) = 0.15, R^2^Y (cum) = 0.974, Q^2^ (cum) = 0.928 in positive mode. The R^2^ and Q^2^ values were R^2^X (cum) = 0.284, R^2^Y (cum) = 0.99, Q^2^ (cum) = 0.764 in negative mode.


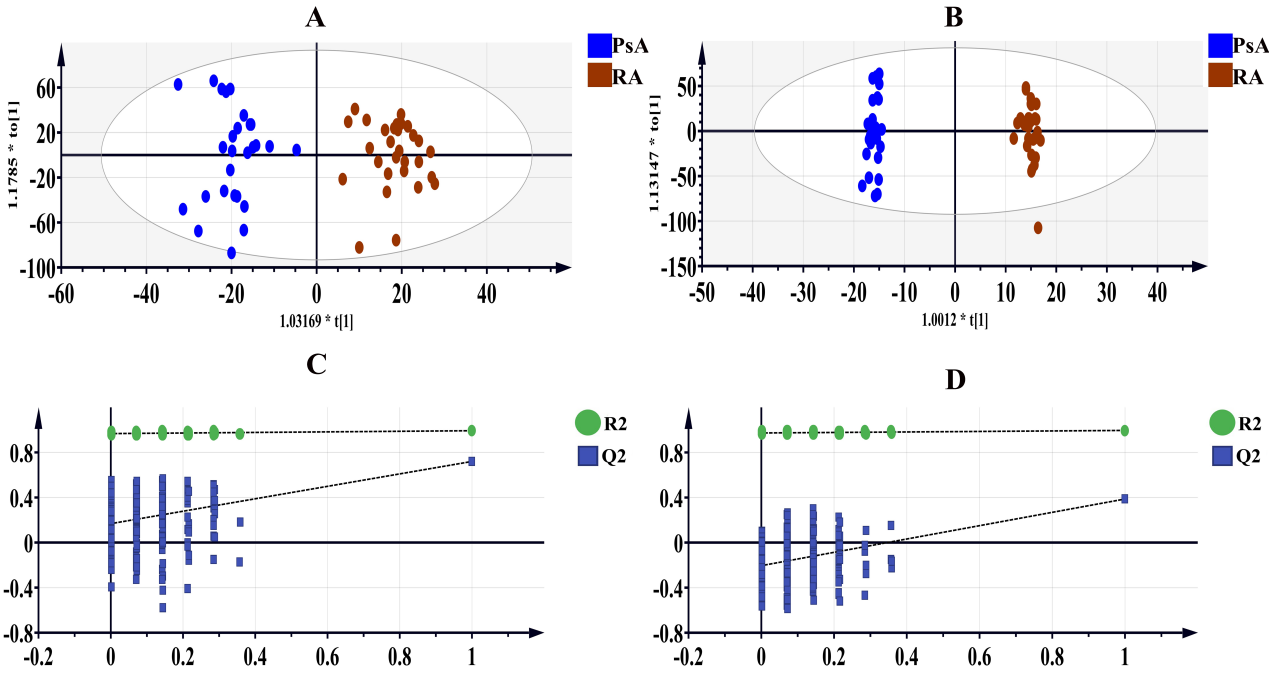


**Supplementary Figure S6.** OPLS-DA score scatter plots of non-polar extracts between PsA group and RA group in positive ion mode (A) and negative ion mode (B). The result of permutation test in positive ion mode (C) and negative ion mode (D). The R^2^ and Q^2^ values were R^2^X (cum) = 0.223, R^2^Y (cum) = 0.938, Q^2^ (cum) = 0.533 in positive mode. The R^2^ and Q^2^ values were R^2^X (cum) = 0.293, R^2^Y (cum) = 0.983, Q^2^ (cum) = 0.169 in negative mode.
